# Morphometric traits predict educational attainment independently of socioeconomic background

**DOI:** 10.1186/s12889-019-8072-7

**Published:** 2019-12-18

**Authors:** Markus Valge, Richard Meitern, Peeter Hõrak

**Affiliations:** Department of Zoology, University of Tartu, Vanemuise 46, 51014 Tartu, Estonia

**Keywords:** Cranial volume, Educational attainment, Face width, Height, Socioeconomic position, Testosterone

## Abstract

**Background:**

Aim of this study is to describe the relationship between anthropometric traits and educational attainment among Estonian schoolchildren born between 1937 and 1962. We asked whether height, cranial volume and face width (a testosterone-dependent trait), measured in childhood predict later educational attainment independently of each other, family socioeconomic position (SEP) and sex. Associations between morphometric traits and education and their interactions with biosocial variables are of scholarly importance because higher education is nearly universally associated with low fertility in women, and often with high fertility in men. Hence, morphometric traits associated with educational attainment are targeted by natural selection and describing the exact nature of these associations is relevant for understanding the current patterns of evolution of human body size.

**Methods:**

Data on morphometric measurements and family background of 11,032 Estonian schoolchildren measured between seven and 19 years of age were obtained from the study performed by Juhan Aul between 1956 and 1969. Ordinal logistic regression was used for testing the effects of morphometric traits, biosocial variables and their interaction on the cumulative probability of obtaining education beyond primary level.

**Results:**

Of biosocial variables, family SEP was the most important determinant of educational attainment, followed by the sex, rural vs urban origin and the number of siblings. No significant interactions with morphometric traits were detected, i.e., within each category of SEP, rural vs urban origin and sex, taller children and those with larger heads and relatively narrower faces were more likely to proceed to secondary and/or tertiary education. The effect of height on education was independent of cranial volume, indicating that taller children did not obtain more educations because their brains were larger than those of shorter children; height *per se* was important.

**Conclusions:**

Our main finding – that adjusting for other morphometric traits and biosocial variables, morphometric traits still robustly predicted educational attainment, is relevant for understanding the current patterns of evolution of human body size. Our findings suggest that fecundity selection acting on educational attainment could be partly responsible for the concurrent selection for smaller stature and cranial volume in women and opposite trends in men.

## Background

Educational attainment is a strong and robust predictor of essential life outcomes including occupational status, happiness, health, and life expectancy [1]. Since the beginning of the twentieth century, educational attainment is also known as the most important predictor of Darwinian fitness, depressing fertility of women nearly universally in both modern and developing societies [2–4].

Positive and historically persistent associations between educational attainment and body dimensions, particularly height and neurocranial volume, have been described in diverse populations [5–9]. Genome-wide analyses and pedigree studies have established that associations between height, head size, educational attainment, cognitive abilities and parental socioeconomic position (SEP) have genetic basis [9–12]. Further, the same genetic variants appear largely responsible for the phenotypic correlations between these variables [13–15].

Such genetic correlations are informative about the past selection pressures that have led to clustering of life-history and behavioural trait values along the fast-slow continuum of the life-history speed. According to the theory of life-history evolution, traits characteristic for slow pace of life – late maturation and reproduction, slow development, high somatic investment into body and brain growth, intensive parental care, low birth rates and long life-span – have coevolved with high intelligence and conscientious personality traits [16–18].

Although consistent with the life-history theory, these findings leave open the question of whether and how environment modifies the conversion of genetic correlations between the educational attainment and morphometric traits into the phenotype. Environmental exposures may either exaggerate or attenuate translation of genetic predispositions into phenotypes [19]. For instance, genetic influences on educational attainment may vary across the context of childhood SEP [14, 20, 21]. Access to education may modify the relationship between height and cognitive function [22]. Further, variation in morphology of different brain structures can mediate the associations between family income and school achievement during adolescence [23] and memory performance in adulthood [24].

Aim of this study is to describe the relationship between three anthropometric traits and educational attainment among the large sample of Estonian schoolchildren born between 1937 and 1962. In particular, we ask whether height, cranial volume and face width, measured in childhood predict later educational attainment independently of each other, family SEP and sex.

These questions are relevant for understanding the aetiology of individual differences in educational attainment that are sensitive to genetic as well as societal factors and growth environment [14, 25, 26]. For instance, although numerous studies have recorded positive associations between cranial volume or height vs educational attainment, only few have simultaneously tested the effect of both [27]. At proximate level, distinguishing the effects of height vs cranial volume on education is important because of strong and sex-specific allometric relationships between these traits [28, 29]. Ultimately, accounting for such allometric relationships between anthropometric traits is also important for predicting the rates and directions of evolution of human phenotype. For instance, the question whether the contribution of height to educational attainment is independent or caused by allometric correlation with cranial volume (a proxy for cognitive ability) is relevant for understanding the evolutionary forces acting on morphometric traits via correlated selection on education [see, e.g., 30].

Another morphometric trait that is interesting in the context of educational attainment is face width, an important component of face masculinity [30, 31]. Face width is sensitive to testosterone exposure in utero [32] and during puberty [30]. Men and women with masculine faces are perceived as more aggressive, dominant and strong reviewed by [33–36]. Associations between adult testosterone levels and face masculinity are less clear [37–39]. Notably, however, a small number of studies have found that facial masculinity correlates with another proxy of circulating testosterone – physical strength among both men [40, 41] and women [35, 42]. It is highly likely that all the above-described associations relate to genetic differences in testosterone production as genetic variants that are associated to higher testosterone levels in the body are also associated with facial masculinity [43].

We had no predictions about the direction of association between facial width and educational attainment. On the one hand, higher fetal testosterone levels may be associated with compromised development of cognitive abilities in early childhood (at that, affecting different components of cognition among boys [44, 45] and girls [46, 47]). On the other hand, facial masculinity in men seems to be commonly associated with success and goal attainment not only in in various competitive social contexts but also in prosaically oriented settings [reviewed by 49]. Endogenous male testosterone positively predicts cognitive performance in both cross-sectional and longitudinal analyses reviewed in [48] and testosterone administration often (but not always) improves visuospatial cognitive function [49]. As regards academic performance, Kausel et al. [50] showed that among both male and female university students, relative face width predicted positively achievement in non-quantitative (i.e., orally examined) courses while grade points earned on the basis of written tests showed no associations with face morphology.

We start with testing the assumption that face width can be considered as a testosterone-dependent trait by examining the correlations between face width and handgrip strength. Next we ask if morphometric traits – height, cranial volume, and face width measured during school years predict the highest educational level the children were going to obtain. Specifically, we aim to test whether previously described associations between height and educational outcomes can be ascribed to allometric relationship between cranial volume (a proxy of cognitive ability) in which case we predicted that inclusion of cranial volume as a predictor variable will cancel the effect of height on educational attainment. Alternatively, if height has an independent effect on education, we expected that both predictors will remain simultaneously significant in the model. Next we test for the occurrence of significant interaction terms between morphometric traits vs sex and two markers of growth conditions – parental SEP and rural vs urban origin. This enables to establish whether the associations between body parameters and education are similar among boys and girls and in children growing up with differential access to material and cognitive resources.

## Methods

Data on morphometric measurements and family background were obtained from the anthropometric study performed by Juhan Aul between 1956 and 1969. The historical background of this sample is described by Hõrak and Valge [51]. Face width was measured as a bizygomatic distance, i.e., maximal distance between the most lateral points on the zygomatic arches. Cranial volume was calculated according to Rushton [52]: 7.884*(head length-11) + (10.842*head width-11)-1593.96 for girls and 6.752*(head length-11) + (11.421*head width-11)-1434.06 for boys (units in mm).

The dataset involves 15,205 girls and 11,757 boys (for both, average age = 12.7, SD = 3.1, range = 7–19 years); this dataset 16 times larger than the one used in the previous study of Hõrak and Valge [51] and involves both girls and boys. These data were used for calculation of age- and sex-specific residuals for height, cranial volume, face width and handgrip strength and correlations between these (Additional file 1: Figure S2-S3). Residuals were calculated with smooth non-parametric regression line and automatic smoothing parameter selection using package ‘gam’ for R [53] and transformed to z-scores within sexes. In addition to age in days, regression included birth date as a predictor variable to account for secular increase in body dimensions during the study period [see 53].

From this sample, we identified a subset of 11,032 individuals whose educational attainment was recorded in the Estonian Population Registry (https://e-estonia.com/solutions/interoperability-services/population-registry/). Data on the highest level of education obtained were based on self-reported data from the last Estonian population census in 2011 (https://www.stat.ee/phc2011); by that time all the subjects had completed their maximum level of education. Educational attainment was divided into three categories: primary (8 years of schooling or less), secondary (including secondary vocational) and tertiary (> 11 years of schooling). On the basis of parental professions recorded during data collection, participants were assigned to parental SEP values (highest in the family) as unskilled manual workers, skilled manual workers or non-manual workers.

Ordinal logistic regression [54] was used for testing the effects of morphometric traits, biosocial variables and their interaction on the cumulative probability of obtaining education beyond primary level. Additionally we run the same models in binary logistic regressions where the dependent variable was either obtaining tertiary vs secondary or secondary vs primary education. All models were tested for the interactions between birth year vs sex, parental SEP, and urban/rural origin to account for confounding secular trends in educational attainment during the study period. All tests are two-tailed with a P-level below 0.05 as a criterion for significance.

## Results

**Fig. 1:**
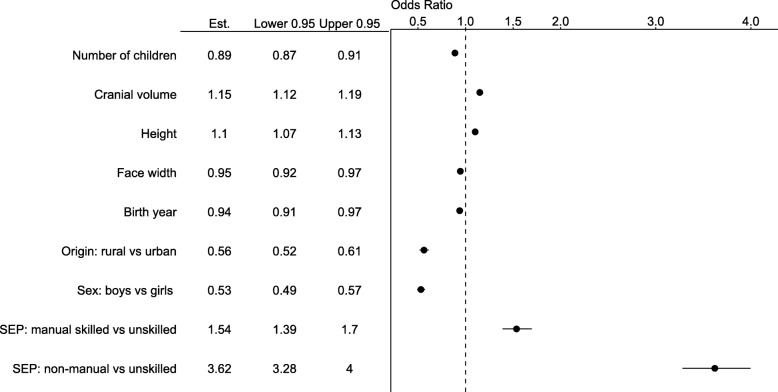
Odds ratios (variable Est.) for testing the main effects of morphometric traits and biosocial variables on the cumulative probability of obtaining education beyond primary level (ordinal logistic regression). See Additional file 1: Table S1 for the estimates and *p*-values of interaction terms. Nagelkerke R^2^ for the model = 0.19

**Fig. 2:**
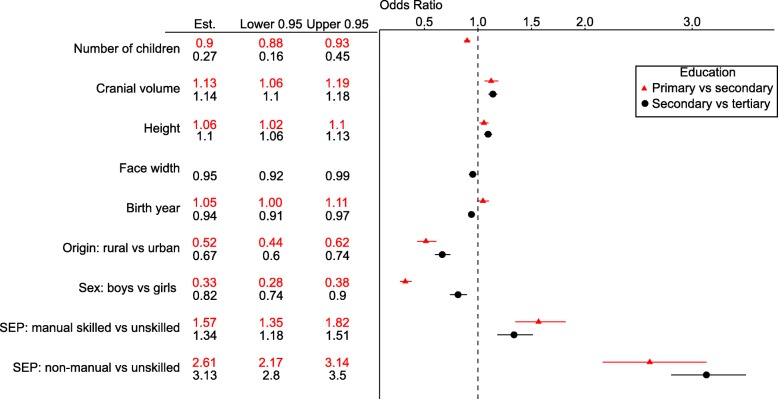
Odds ratios (variable Est.) for testing the main effects of morphometric traits and biosocial variables on the probability of obtaining tertiary vs secondary (Nagelkerke R2 = 0.13) or secondary vs primary (Nagelkerke R^2^ = 0.16) education in binary logistic regression. See Additional file 1: Tables S2-S3 for the estimates and p-values for interaction terms

Higher levels of education were more prevalent among girls and in general, children of urban origin (Additional file 1: Figure S1, Figs. 1-2). All anthropometric variables were positively correlated (Additional file 1: Figure S2-S3). Face width predicted handgrip strength better (girls: r = 0.26; boys: r = 0.35) than another measure of head size, cranial volume (girls: r = 0.20; boys: r = 0.27), confirming our assumption that face width can be regarded as a marker of testosterone exposure.

**Fig. 3:**
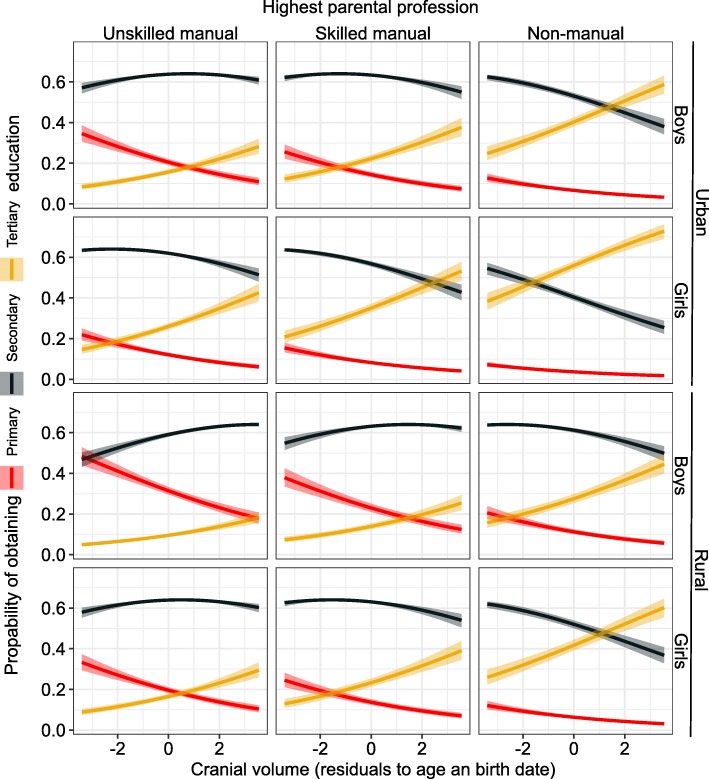
Predicted probabilities ±95% CI of obtaining primary, secondary or tertiary education in relation to cranial volume. Based on the model described in Additional file 1: Table S1 and Figure 1

**Fig. 4:**
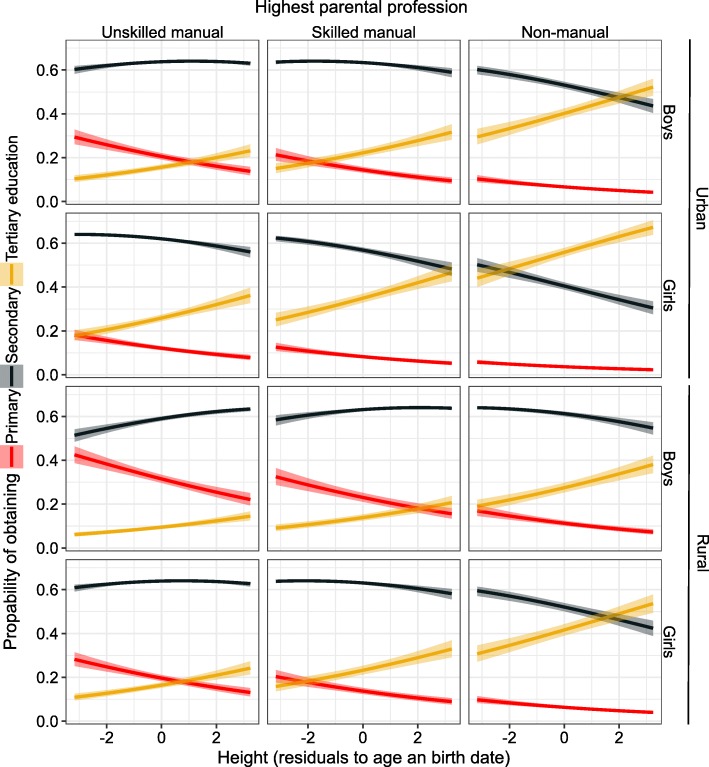
Predicted probabilities ±95% CI of obtaining primary, secondary or tertiary education in relation to height. Based on the model described in Additional file 1: Table S1 and Figure 1

**Fig. 5:**
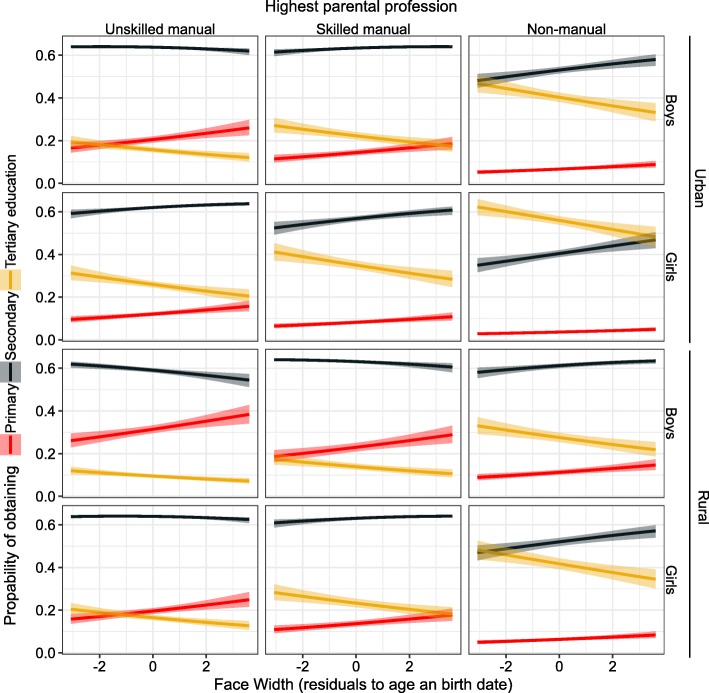
Predicted probabilities ±95% CI of obtaining primary, secondary or tertiary education in relation to face width. Based on the model described in Additional file 1: Table S1 and Figure 1

Parental SEP was the strongest predictor of educational attainment (Figs. 1-4, Additional file 1: Table S1). Yet after adjusting for the covariates in the model, all three anthropometric parameters independently predicted the educational attainment that children were going to obtain. Taller children with larger heads and narrower faces had higher cumulative probability of obtaining either secondary or tertiary education (Figs 3, 4 and 5). None of the interaction terms between morphological variables and factors (sex, rural/urban, SEP) improved the fit of the model in Additional file 1: Table S1 significantly (*p* = 0.13–0.81; likelihood ratio tests).

To illustrate the results, we apply the odds ratios presented in Fig. 1 to the quantile values of raw measurements among the most common age class, 13-years old children (1577 girls and 1276 boys). For girls, being 4.5 cm (2.8%) taller than the median of 155 cm (5. 2 cm above 153 cm for boys; 3.3%) increased the chances of proceeding from primary to secondary or secondary to tertiary education by 10%. Girls with cranial volume 54 cm^3^ (4.1%) above the median of 1262 cm^3^ (boys: 52 cm^3^ above 1367 cm^3^; 3.6%) had 15% higher chances of obtaining the secondary (over primary) or tertiary (over secondary) education. Having a 0.3 cm (2.3%) narrower face than the median of 13 cm increased the chance for proceeding to secondary or tertiary education by 5% for both sexes.

Next we tested in binary logistic analysis whether the same variables as in Fig. 1 predicted the chances of proceeding from secondary to tertiary education. The best model was almost identical to that in Additional file 1: Table S1 with an exception that three-way interaction between sex, urban/rural origin and year of birth (YOB) has lost its significance (Additional file 1: Table S2).

Examination of model predicting the chances of proceeding from primary to secondary education revealed that face width was not significant any more (*P* = 0.063 in Wald test). The best model (Additional file 1: Table S3) also suggested that the effect of cranial volume on proceeding to the secondary education depended on sex. Analysing the data for boys and girls separately revealed that in the case of boys, none of the morphometric variables predicted education (cranial volume: OR = 1.04, 95% CI = 0.99–1.10; height: OR = 1.05, 95% CI = 0.99–1.10). In the case of girls, both morphological variables remained significant although notably, the confidence intervals showed some overlap with these of boys (cranial volume: OR = 1.13, 95% CI = 1.06–1.19; height: OR = 1.07, 95% CI = 1.01–1.14).

## Discussion

Parental SEP was the most important determinant of educational attainment. Children whose parents were in non-manual profession had 3.6 times higher and children whose parents were in skilled manual profession had 1.5 times higher cumulative odds of obtaining educational level beyond primary (Fig. 1). This is consistent with previous findings demonstrating that parental SEP (besides intelligence) is the most important predictor of educational attainment worldwide [55, 56], including Estonia [57]. The mechanism of this connection may involve both social and genetic inheritance of both SEP and education [14] and the genetic correlations between the two are not trivial. For instance, social deprivation can genetically strongly correlate with educational attainment (r_g_ = − 0.55 in UK Biobank sample [58];).

Being of rural (vs urban) origin reduced the odds of obtaining education beyond primary level by 44% and being a boy vs girl by 47%. Rural-urban educational gap is consistent with the global evidence [59]. However, the higher female to male ratio in secondary and tertiary education seems to be specific peculiarity of some Soviet and socialist regimes [60] as compared to the global patterns in the middle of the twentieth century [61]. Figures 1 and 2 also indicate that after accounting for socioeconomic and sex differences, number of siblings had still independent (though small) effect on education. This pattern, too, is consistent with previous evidence e.g., [62].

After adjusting for biosocial variables, all morphometric traits still significantly predicted educational attainment. Thus, within each category of parental SEP, rural vs urban origin and sex, taller children and those with larger heads and narrower faces were more likely to proceed to secondary and/or tertiary education. Assuming that measured morphometric traits reflect some intrinsic qualities of individuals, our results provide anthropometric evidence that within socioeconomic strata, a detectable meritocracy in the Estonian educational system under the Soviet regime existed. Our assumption that cranial volume and height reflect qualities related to educational achievement is supported by previous findings from the Juhan Aul’s dataset, showing that girls progressing slowly at school (those that were > 1.5 years old for their grade) were shorter and had smaller heads than those progressing at school at an appropriate rate for their age [63].

### Cranial volume

As regards cranial volume, the most parsimonious explanation to its independent effect on education is its strong correlation with brain size (head circumference vs brain volume: r = 0.72–0.86 [64]; head circumference vs intracranial volume: r = 0.91 [9]). Brain and head size, in turn, correlate phenotypically with intelligence (r = 0.19–0.63 [27, 29, 65, 66]). Cranial volume also correlates genetically with IQ (r_g_ = 0.25 [67]; r_g_ = 0.27 [9] and educational attainment (r_g_ = 0.14–0.44 [9, 68]). Genetic correlations between IQ and educational attainment are even stronger (r_g_ = 0.70–0.73 [69, 70]). Cranial volume is sensitive to resource availability during growth too [28, 51]. Again, this association can have a strong genetic component (r_g_ between household income and intracranial volume is 0.53 in UK Biobank sample [58]).

Our finding that cranial volume predicted educational attainment (controlling for social stratification and height) is similar to that of Nave et al. [27] who assessed the brain volume on the basis of MRI scans of 13,608 40–69 year old participants of UK Biobank. The similarity of the findings to these two large-scale studies indicates that head size is a robust predictor of educational attainment, irrespective of the method of measurement and whether using adult population experiencing market economy or children pursuing education under the Soviet regime. Nave et al. [27] did not find any sex differences in associations between brain volume and cognitive ability or educational attainment. Results of our study are generally consistent with this finding with an exception that odds of obtaining secondary education depended on head size among girls but not in boys.

### Height

Our study confirms earlier findings that height is positively associated with educational attainment [6, 71, 72]. One of the likely reasons for this association is genetic correlation (r_g_ = 0.16–0.65, depending on age) between height and IQ [73]. This may occur either because the sets of genes affecting these traits partially overlap (pleiotropy) and/or because positive assortative mating for height and education (as components of overall attractiveness) causes cross-trait assortative mating, leading to genetic covariation due to gametic phase disequilibrium [10].

Importantly, this study also shows that the effect of height on education was independent of cranial volume. This means that taller children did not obtain more educations because their brains were larger than those of shorter children; height per se was important [see 6]. One possible explanation for this independent height effect is that taller children possess more non-cognitive skills. For instance taller children may more likely participate in sport activities that enable to accumulate social skills that favour continuation of education [72]. It is also possible that active gene–environment correlation may play a role, for example, if tall children generate intellectually more stimulating environments than short children [73]. Yet another, not mutually exclusive explanation would be discrimination or social stigma against shorter individuals. For instance Vågerö and Modin [74] shoved that among Swedish men with similar cognitive abilities; those who were taller were more likely to obtain university education than shorter ones. Lastly, the finding that the effect of height on education was independent of cranial volume is consistent with an idea that variation in these traits reflects genome-wide mutational loads. I.e., height, cranial volume and educational attainment may stand as independent markers of overall genetic quality see [10, 75, 76].

### Face width

We a found a small effect of face width on the odds of obtaining tertiary education. Although face width correlated positively with height and cranial volume, children with narrower faces (for fixed height and cranial volume) had higher odds for continuation of education beyond the secondary level. Assuming that face width is a proxy for testosterone exposure in utero and/or during adolescence, our results suggest that both boys and girls with higher testosterone exposure or levels were less likely to progress on educational path.

The result that both boys and girls with narrower, less masculine faces were more likely to obtain higher education is notable as it contrasts with previous finding that both male and female university students with relatively wider faces gained better grades in orally examined courses [50] and that face masculinity predicts success in various competitive and pro-socially oriented settings [77]. Our finding is also not compatible with studies showing the positive association between circulating testosterone levels and cognitive abilities (see Introduction). On the other hand, assuming that face width is testosterone-dependent trait, our results compare favourably with those of a study of 4462 US military veterans, whose serum testosterone levels correlated negatively with the years of education (r = − 0.11) and intelligence (r = − 0.07) [78]. Mediation analysis of veteran data showed that the effects of testosterone on education were mediated similarly by high antisocial behaviour and low intelligence. Dabbs Jr. [78] suggests that characteristic interests could lead high-testosterone individuals away from school and towards a world of action: given that testosterone is related to simple perseverant responding, “high-testosterone individuals may find little satisfaction spending hours sitting in classrooms and considering ideas”. If such reasoning is correct then proposed relationship between high testosterone and low perseverance might also explain our finding that children with narrower faces were more likely to pursue tertiary education. Consistent with such an explanation are several studies showing that high levels of testosterone are linked to higher impulsivity and weaker behavioural control reviewed in [79], i.e., traits characteristic to the fast life histories and low educational attainment [80].

### Strengths and limitations

Current study is the second largest one after that of Nave and co-authors [27], showing that cranial dimensions and height predict the educational attainment, controlling for the biosocial background. Its strengths are that due to historical reasons, the participation was not voluntary, which considerably reduces the recruitment selection and that all the participants were younger than 20, which almost entirely eliminates the mortality selection in the sample see [81]. For instance, the sample of Nave et al. [27] consisted almost entirely of adults and elderly that overrepresented individuals of higher SEP.

On the other hand, measuring children poses a limitation because they grow at individually different rates. E.g., a child might be small for age at age 13 but still end up in above average size if his/her pubertal growth spurt starts later than average and vice versa. This is an unavoidable problem of all cross-sectional morphometric studies of children; however such measurement errors are conservative as they do not introduce any systematic bias. Another limitation is that the study was based on secondary data, thus its design was not under the control of the authors. Nevertheless, we consider that the quality, uniqueness and richness of the dataset collected by Prof Juhan Aul weighs up against possible shortcomings of the study design (e.g., randomization issues). Importantly for quality, all the morphometric measurements were collected by the same person and recording of sufficient amount of background variables enables statistically control for the main biosocial confounders.

## Conclusions and implications

Our main finding – that adjusting for other morphometric traits and biosocial variables, morphometric traits still robustly predicted educational attainment, is relevant for understanding the current patterns of evolution of human body size. Across western populations, female education is almost universally negatively related to reproductive success [82–84] while among men, typically stabilizing or positive selection on education prevails [reviewed in 84, 85, 86]. According to the census data, Estonian women with only primary education bore 0.5 to 0.75 more children on average than women with tertiary education throughout the twentieth century [85]. In parallel, natural selection in developed countries usually favours shorter women and taller men [86–89]. These parallel patterns refer to the possibility that selection acting on educational attainment could be at least partly responsible for the concurrent selection for smaller stature and cranial volume in women and opposite trends in men. So far the phenotypic response to this correlated selection has been masked by secular increase in body dimensions [see 53]. However, one might predict that with general improvement and equalisation of growth conditions, genetic between-individual differences in body dimensions will become more clearly detectable in the phenotype [see also 82, 86, 89].

That evolutionary changes can be rapid is confirmed by a study in Iceland, showing that selection against genetic variants associated with educational attainment can lead to change in the genetic composition of population in few generations [90]. Hence, the possibility that traits genetically correlated with educational attainment (such as height and cranial volume) will show a correlated response in the phenotype in the future generations, should not be underestimated. At the same time, the effect of education on reproduction involves a substantial non-genetic component too [14, 26]. In this context, our findings suggest that patterns of current selection on human body dimensions constitute an example of gene-culture coevolution.

As for practical concerns, the finding of this study that height predicts educational attainment independently of cranial volume, sex and SEP calls for the research on possible issues of discrimination or social stigma against shorter individuals at school. Behavioural pathways (and associated genetic underpinnings) that lead to educational advantage of taller children deserve further investigation.

## Supplementary information

**Additional file 1.** Electronic supplementary material ESM 1.

**Additional file 2.** Electronic supplement 2.

## Data Availability

The dataset supporting the conclusions of this article is available in the https://cran.r-project.org/package=ormPlot. Commented R codes for statistical analyses (including tests for the proportional odds assumption) and graphics have been uploaded in ESM 2 (Additional file 2).

## Abbreviations

CI: Confidence interval
IQ: Intelligence quotient
SEP: Socioeconomic position
YOB: Year of birth

## References

[1] Deary IJ, Harris SE, Hill WD (2019). What genome-wide association studies reveal about the association between intelligence and physical health, illness, and mortality. Curr Opin Psychol.

[2] Skirbekk V (2008). Fertility trends by social status. Demogr Res.

[3] Wang M, Fuerst J, Ren J (2016). Evidence of dysgenic fertility in China. Intelligence.

[4] Meisenberg G (2019). Social and reproductive success in the United States: the roles of income, education and cognition. The Mankind Quarterley.

[5] Silventoinen K (2003). Determinants of variation in adult body height. J Biosoc Sci.

[6] Magnusson PKE, Rasmussen F, Gyllensten UB (2006). Height at age 18 years is a strong predictor of attained education later in life: cohort study of over 950 000 Swedish men. Int J Epidemiol.

[7] Li H, DiGirolamo AM, Barnhart HX, Stein AD, Martorell R (2004). Relative importance of birth size and postnatal growth for women's educational achievement. Early Hum Dev.

[8] Ivanovic DM, Valenzuela RB, Almagià AF, Barrera CR, Arancibia VC, Larraín CG, Silva CFA, Billeke PB, Zamorano FM, Villagrán FS (2019). Impact of anthropometric nutritional parameters on the university selection test in Chile: a multifactorial approach. Nutrition.

[9] Haworth S, Shapland CY, Hayward C, Prins BP, Felix JF, Medina-Gomez C, Rivadeneira F, Wang C, Ahluwalia TS, Vrijheid M (2019). Low-frequency variation in TP53 has large effects on head circumference and intracranial volume. Nat Commun.

[10] Keller MC, Garver-Apgar CE, Wright MJ, Martin NG, Corley RP, Stallings MC, Hewitt JK, Zietsch BP (2013). The genetic correlation between height and IQ: shared genes or Assortative mating?. PLoS Genet.

[11] Marioni RE, Davies G, Hayward C, Liewald D, Kerr SM, Campbell A, Luciano M, Smith BH, Padmanabhan S, Hocking LJ (2014). Molecular genetic contributions to socioeconomic status and intelligence. Intelligence.

[12] Marioni RE, Batty GD, Hayward C, Kerr SM, Campbell A, Hocking LJ, Porteous DJ, Visscher PM, Deary IJ (2014). Common genetic variants explain the majority of the correlation between height and intelligence: the generation Scotland study. Behav Genet.

[13] Plomin R, Deary IJ (2015). Genetics and intelligence differences: five special findings. Mol Psychiatry.

[14] Belsky DW, Domingue BW, Wedow R, Arseneault L, Boardman JD, Caspi A, Conley D, Fletcher JM, Freese J, Herd P (2018). Genetic analysis of social-class mobility in five longitudinal studies. Proc Natl Acad Sci.

[15] Trzaskowski Maciej, Harlaar Nicole, Arden Rosalind, Krapohl Eva, Rimfeld Kaili, McMillan Andrew, Dale Philip S., Plomin Robert (2014). Genetic influence on family socioeconomic status and children's intelligence. Intelligence.

[16] Figueredo AJ, Cabeza de Baca T, Woodley MA (2013). The measurement of human life history strategy. Personal Individ Differ.

[17] Rushton JP (1985). Differential K theory: the sociobiology of individual and group differences. Personal Individ Differ.

[18] Sefcek JA, Figueredo AJ (2010). A life-history model of human fitness indicators. Biodemography Soc Biol.

[19] Rutter M, Moffitt TE, Caspi A (2006). Gene–environment interplay and psychopathology: multiple varieties but real effects. J Child Psychol Psychiatry.

[20] Tucker-Drob EM, Bates TC (2016). Large cross-national differences in gene × socioeconomic status interaction on intelligence. Psychol Sci.

[21] Turkheimer E, Haley A, Waldron M, D'Onofrio B, Gottesman II (2003). Socioeconomic status modifies heritability of IQ in young children. Psychol Sci.

[22] Kobayashi LC, Berkman LF, Wagner RG, Kahn K, Tollman S, Subramanian S (2019). Education modifies the relationship between height and cognitive function in a cross-sectional population-based study of older adults in rural South Africa. Eur J Epidemiol.

[23] Brito NH, Piccolo LR, Noble KG (2017). Associations between cortical thickness and neurocognitive skills during childhood vary by family socioeconomic factors. Brain Cogn.

[24] Duval ER, Garfinkel SN, Swain JE, Evans GW, Blackburn EK, Angstadt M, Sripada CS, Liberzon I (2017). Childhood poverty is associated with altered hippocampal function and visuospatial memory in adulthood. Dev Cogn Neurosci.

[25] Rimfeld K, Krapohl E, Trzaskowski M, Coleman JRI, Selzam S, Dale PS, Esko T, Metspalu A, Plomin R (2018). Genetic influence on social outcomes during and after the soviet era in Estonia. Nat Hum Behav.

[26] Conley D, Domingue BW, Cesarini D, Dawes C, Rietveld CA, Boardman JD (2015). Is the effect of parental education on offspring biased or moderated by genotype?. Sociol Sci.

[27] Nave G, Jung WH, Karlsson Linnér R, Kable JW, Koellinger PD (2019). Are bigger brains smarter? Evidence from a large-scale preregistered study. Psychol Sci.

[28] Kim YS, Park IS, Kim HJ, Kim D, Lee NJ, Rhyu IJ (2018). Changes in intracranial volume and cranial shape in modern Koreans over four decades. Am J Phys Anthropol.

[29] Rushton JP, Ankney CD (2009). Whole brain size and general mental ability: a review. Int J Neurosci.

[30] Marečková K, Weinbrand Z, Chakravarty MM, Lawrence C, Aleong R, Leonard G, Perron M, Pike GB, Richer L, Veillette S (2011). Testosterone-mediated sex differences in the face shape during adolescence: subjective impressions and objective features. Horm Behav.

[31] Hodges-Simeon CR, Hanson Sobraske KN, Samore T, Gurven M, Gaulin SJC (2016). Facial width-to-height ratio (fWHR) is not associated with adolescent testosterone levels. PLoS One.

[32] Whitehouse AJO, Gilani SZ, Shafait F, Mian A, Tan DW, Maybery MT, Keelan JA, Hart R, Handelsman DJ, Goonawardene M (2015). Prenatal testosterone exposure is related to sexually dimorphic facial morphology in adulthood. Proc R Soc B Biol Sci.

[33] Quist MC, Watkins CD, Smith FG, DeBruine LM, Jones BC (2011). Facial masculinity is a cue to women’s dominance. Personal Individ Differ.

[34] Palmer-Hague JL, Watson NV (2016). Effects of mother and father dominance on offspring sex in contemporary humans. Adapt Hum Behav Physiol.

[35] Hahn AC, Holzleitner IJ, Lee AJ, Kandrik M, O'Shea KJ, DeBruine LM, Jones BC (2019). Facial masculinity is only weakly correlated with handgrip strength in young adult women. Am J Hum Biol.

[36] Costa M, Lio G, Gomez A, Sirigu A (2017). How components of facial width to height ratio differently contribute to the perception of social traits. PLoS One.

[37] Lefevre CE, Lewis GJ, Perrett DI, Penke L (2013). Telling facial metrics: facial width is associated with testosterone levels in men. Evol Hum Behav.

[38] Eisenbruch AB, Lukaszewski AW, Simmons ZL, Arai S, Roney JR (2018). Why the wide face? Androgen receptor gene polymorphism does not predict men’s facial width-to-height ratio. Adapt Hum Behav Physiol.

[39] Pound N, Penton-Voak IS, Surridge AK (2009). Testosterone responses to competition in men are related to facial masculinity. Proc R Soc B Biol Sci.

[40] Bernhard Fink NNHS (2007). Male facial appearance signals physical strength to women. Am J Hum Biol.

[41] Windhager S, Schaefer K, Fink B (2011). Geometric morphometrics of male facial shape in relation to physical strength and perceived attractiveness, dominance, and masculinity. Am J Hum Biol.

[42] Van Dongen S (2014). Associations among facial masculinity, physical strength, fluctuating asymmetry and attractiveness in young men and women. Ann Hum Biol.

[43] Roosenboom J, Indencleef K, Lee MK, Hoskens H, White JD, Liu D, Hecht JT, Wehby GL, Moreno LM, Hodges-Simeon C (2018). SNPs associated with testosterone levels influence human facial morphology. Front Genet.

[44] Hollier LP, Mattes E, Maybery MT, Keelan JA, Hickey M, Whitehouse AJO (2013). The association between perinatal testosterone concentration and early vocabulary development: a prospective cohort study. Biol Psychol.

[45] Farrant BM, Mattes E, Keelan JA, Hickey M, Whitehouse AJO (2013). Fetal testosterone, socio-emotional engagement and language development. Infant Child Dev.

[46] Finegan J-AK, Niccols GA, Sitarenios G (1992). Relations between prenatal testosterone levels and cognitive abilities at 4 years. Dev Psychol.

[47] Jacklin CN, Wilcox KT, Maccoby EE (1988). Neonatal sex-steroid hormones and cognitive abilities at six years. Dev Psychobiol.

[48] Trumble BC, Stieglitz J, Thompson ME, Fuerstenberg E, Kaplan H, Gurven M. Testosterone and male cognitive performance in Tsimane forager-horticulturalists. Am J Hum Biol. 2014. 10.1002/ajhb.22665 PMC8446946 25429990

[49] Toivainen T, Pannini G, Papageorgiou KA, Malanchini M, Rimfeld K, Shakeshaft N, Kovas Y (2018). Prenatal testosterone does not explain sex differences in spatial ability. Sci Rep.

[50] Kausel EE, Ventura S, Datawheel L, Díaz D, Vicencio F (2018). Does facial structure predict academic performance?. Personal Individ Differ.

[51] Hõrak P, Valge M. Why did children grow so well at hard times? The ultimate importance of pathogen control during puberty. Evolution, Medicine, and Public Health. 2015:167–78. 10.1093/emph/eov017 PMC4530472 26198188

[52] Rushton JP (1997). Race, evolution, and behavior: a life history perspective.

[53] Hastie T: Package ‘gam': Generalized Additive Models. R package version 1.16. In*.*; 2018.

[54] Harrell Jr F: rms: Regression Modeling Strategies. R package version 5.1–3.1. In*.*; 2019.

[55] Strenze T (2007). Intelligence and socioeconomic success: a meta-analytic review of longitudinal research. Intelligence.

[56] Keage HAD, Muniz G, Kurylowicz L, Van Hooff M, Clark L, Searle AK, Sawyer MG, Baghurst P, McFarlane A (2016). Age 7 intelligence and paternal education appear best predictors of educational attainment: the Port Pirie cohort study. Aust J Psychol.

[57] Saar E, Aimre K-A: Unequal educational transitions in Estonia: tracking and family background. In. Tallinna Ülikool; 2012. https://www.digar.ee/arhiiv/nlib-digar:196880

[58] Hill WD, Hagenaars SP, Marioni RE, Harris SE, Liewald DCM, Davies G, Okbay A, McIntosh AM, Gale CR, Deary IJ (2016). Molecular genetic contributions to social deprivation and household income in UK biobank. CB.

[59] Ulubaşoğlu MA, Cardak BA (2007). International comparisons of rural–urban educational attainment: data and determinants. Eur Econ Rev.

[60] Titma M, Saar E (1995). Regional differences in soviet secondary education. Eur Sociol Rev.

[61] Barro RJ, Lee JW (2013). A new data set of educational attainment in the world, 1950–2010. J Dev Econ.

[62] Lawson DW, Makoli A, Goodman A (2013). Sibling configuration predicts individual and descendant socioeconomic success in a modern post-industrial society. PLoS One.

[63] Hõrak P, Valge M (2016). Old-for-grade girls reproduce but do not mature early: simply a mechanistic link between educational progress and pace of life?. Intelligence.

[64] Ivanovic DM, Leiva BP, Castro CG, Olivares MG, Jansana JMM, Castro VG, Almagià AAF, Toro TD, Urrutia MSC, Miller PT (2004). Brain development parameters and intelligence in Chilean high school graduates. Intelligence.

[65] Ritchie SJ, Booth T, MdC VH, Corley J, Maniega SM, Gow AJ, Royle NA, Pattie a, Karama S, Starr JM (2015). Beyond a bigger brain: multivariable structural brain imaging and intelligence. Intelligence.

[66] Gignac GE, Bates TC (2017). Brain volume and intelligence: The moderating role of intelligence measurement quality. Intelligence.

[67] Savage JE, Jansen PR, Stringer S, Watanabe K, Bryois J, de Leeuw CA, Nagel M, Awasthi S, Barr PB, Coleman JRI (2018). Genome-wide association meta-analysis in 269,867 individuals identifies new genetic and functional links to intelligence. Nat Genet.

[68] Hagenaars SP, Harris SE, Davies G, Hill WD, Liewald DCM, Ritchie SJ, Marioni RE, Fawns-Ritchie C, Cullen B, Malik R, et al. Shared genetic aetiology between cognitive functions and physical and mental health in UK biobank (N=112151) and 24 GWAS consortia. Mol Psychiatry. 2016. 10.1038/mp.2015.225 PMC5078856 26809841

[69] Davies G, Marioni RE, Liewald DC, Hill WD, Hagenaars SP, Harris SE, Ritchie SJ, Luciano M, Fawns-Ritchie C, Lyall D, et al. Genome-wide association study of cognitive functions and educational attainment in UK biobank (N=112[thinsp]151). Mol Psychiatry. 2016. 10.1038/mp.2016.45 PMC4879186 27046643

[70] Hill WD, Marioni RE, Maghzian O, Ritchie SJ, Hagenaars SP, McIntosh AM, Gale CR, Davies G, Deary IJ. A combined analysis of genetically correlated traits identifies 187 loci and a role for neurogenesis and myelination in intelligence. Mol Psychiatry. 2018. 10.1038/s41380-017-0001-5 PMC6344370 29326435

[71] Huang Y, van Poppel F, Lumey LH (2015). Differences in height by education among 371,105 Dutch military conscripts. Econ Human Biol.

[72] Cinnirella F, Piopiunik M, Winter J (2011). Why does height matter for educational attainment? Evidence from German children. Econ Human Biol.

[73] Silventoinen K, Posthuma D, van Beijsterveldt T, Bartels M, Boomsma DI (2006). Genetic contributions to the association between height and intelligence: evidence from Dutch twin data from childhood to middle age. Genes Brain Behav.

[74] Vågerö D, Modin B (2006). Commentary: the associations between height, cognition, and education and their relevance for health studies. Int J Epidemiol.

[75] Arden R, Gottfredson LS, Miller G (2009). Does a fitness factor contribute to the association between intelligence and health outcomes? Evidence from medical abnormality counts among 3654 US veterans. Intelligence.

[76] Houle D (2000). Is there a g factor for fitness?. Nat Intell.

[77] Hahn T, Winter NR, Anderl C, Notebaert K, Wuttke AM, Clément CC, Windmann S (2017). Facial width-to-height ratio differs by social rank across organizations, countries, and value systems. PLoS One.

[78] Dabbs JM (1992). Testosterone and occupational achievement. Soc Forces.

[79] Doi H, Nishitani S, Shinohara K (2015). Sex difference in the relationship between salivary testosterone and inter-temporal choice. Horm Behav.

[80] Del Giudice M (2014). An evolutionary life history framework for psychopathology. Psychol Inq.

[81] Hõrak P, Valge M, Fischer K, Mägi R, Kaart T (2019). Parents of early maturing girls die younger. Evol Appl.

[82] Stulp G, Verhulst S, Pollet TV, Buunk AP (2012). The effect of female height on reproductive success is negative in western populations, but more variable in non-western populations. Am J Hum Biol.

[83] Byars SG, Ewbank D, Govindaraju DR, Stearns SC (2010). Natural selection in a contemporary human population. Proc Natl Acad Sci.

[84] Stearns SC, Govindaraju DR, Ewbank D, Byars SG. Constraints on the coevolution of contemporary human males and females. Proc Biol Sci. 2012. 10.1098/rspb.2012.2024 PMC3497102 23034705

[85] Tiit E-M: Marriage and childbirth trends. In: Census snapshots. edn. Tallinn: Statistics Estonia; 2013: 32–38. https://www.stat.ee/publication-2013_census-snapshots

[86] Stulp Gert, Barrett Louise (2016). Wealth, fertility and adaptive behaviour in industrial populations. Philosophical Transactions of the Royal Society B: Biological Sciences.

[87] Stulp Gert, Barrett Louise, Tropf Felix C., Mills Melinda (2015). Does natural selection favour taller stature among the tallest people on earth?. Proceedings of the Royal Society B: Biological Sciences.

[88] Stearns SC, Byars SG, Govindaraju DR, Ewbank D (2010). Measuring selection in contemporary human populations. Nat Rev Genet.

[89] Beauchamp JP (2016). Genetic evidence for natural selection in humans in the contemporary United States. Proc Natl Acad Sci.

[90] Kong A, Frigge ML, Thorleifsson G, Stefansson H, Young AI, Zink F, Jonsdottir GA, Okbay A, Sulem P, Masson G (2017). Selection against variants in the genome associated with educational attainment. Proc Natl Acad Sci.

