# Supplementary material for: Morphometric traits predict educational attainment independently of socioeconomic background
Source: BMC Public Health. 2019 Dec 18;19:1696. doi: 10.1186/s12889-019-8072-7 (PMC6921596; doi:10.1186/s12889-019-8072-7)
Supplement: Supplementary file 1 — Additional file 1. Electronic supplementary material ESM 1. [file 12889_2019_8072_MOESM1_ESM.pdf]

Electronic supplementary material ESM 1.

Table S1. Summary statistics of the most parsimonious ordinal logistic regression model (log of cumulative odds of obtaining education level beyond primary; see Fig. 1 for the odds ratios and CI-s of the main effects).

Parameter	Estimate	SE	Wald's z	p
Intercept 1	1.576	0.0573	27.52	<0.0001
Intercept 2	-1.4582	0.0568	-25.66	<0.0001
Rural vs Urban	-0.5591	0.0428	-13.05	<0.0001
Girls vs Boys	0.637	0.0398	16.02	<0.0001
manual skilled vs unskilled	0.4294	0.0509	8.43	<0.0001
non-manual vs unskilled	1.2867	0.0502	25.61	<0.0001
Number of children	-0.1177	0.0127	-9.28	<0.0001
Cranial volume	0.2102	0.0232	9.05	<0.0001
Height	0.1493	0.0215	6.95	<0.0001
Face width	-0.0851	0.0237	-3.6	0.0003
Birth year	-0.0426	0.008	-5.32	<0.0001
Girls * Birth year	0.0215	0.0088	2.45	0.0141
Rural * Birth year	0.0646	0.0094	6.91	<0.0001

Table S2. Summary statistics of the most parsimonious binary logistic regression model (log odds of obtaining tertiary vs secondary education; see Fig. 2 for the odds ratios and CI-s of the main effects).

Parameter	Estimate	SE	Wald's z	p
Intercept	-1.0141	0.072	-14.09	<0.0001
Rural vs Urban	-0.404	0.0552	-7.32	<0.0001
Girls vs Boys	0.2172	0.05	4.34	<0.0001
manual skilled vs unskilled	0.2909	0.0633	4.59	<0.0001
non-manual vs unskilled	1.1422	0.0567	20.13	<0.0001
Number of children	-0.0933	0.0182	-5.12	<0.0001
Cranial volume	0.2055	0.0289	7.11	<0.0001
Height	0.1401	0.0266	5.27	<0.0001
Face width	-0.0759	0.0294	-2.58	0.0099
Birth year	-0.0773	0.0092	-8.41	<0.0001
Girls*Birth year	0.0568	0.0107	5.3	<0.0001

Table S3. Summary statistics of the most parsimonious binary logistic regression model (log odds of obtaining secondary vs primary education; see Fig. 2 for the odds ratios and CI-s of the main effects).

Parameter	Estimate	SE	Wald's z	p
Intercept	1.037	0.0766	13.54	<0.0001
Rural vs Urban	-0.3626	0.0767	-4.73	<0.0001
Girls vs Boys	1.1213	0.0841	13.33	<0.0001
manual skilled vs unskilled	0.451	0.0763	5.91	<0.0001
non-manual vs unskilled	0.9587	0.0944	10.15	<0.0001
Number of children	-0.1018	0.0161	-6.34	<0.0001
Cranial volume	0.0607	0.0388	1.57	0.1175
Height	0.0899	0.0306	2.93	0.0033
Birth year	0.0166	0.0087	1.9	0.0571
Rural*Birth year	0.0569	0.0131	4.35	<0.0001
Rural*Girls	-0.2815	0.1142	-2.46	0.0137
Girls*Cranial volume	0.1202	0.0572	2.1	0.0356

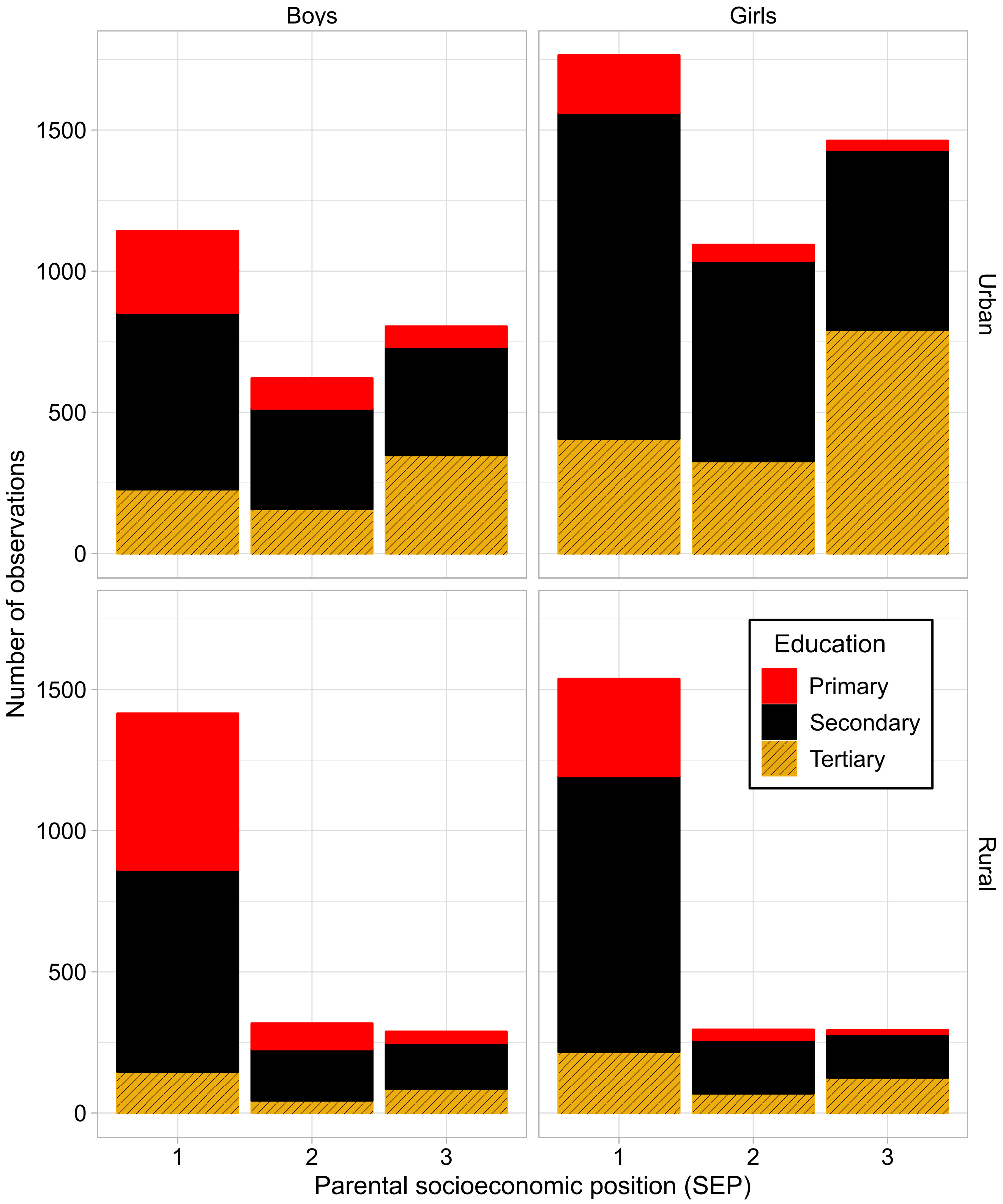

Figure S1. Frequency distribution of educational attainment according to the sex, urban/rural origin and parental SEP. N= 1840 for primary, 6250 for secondary and 2942 for tertiary education.

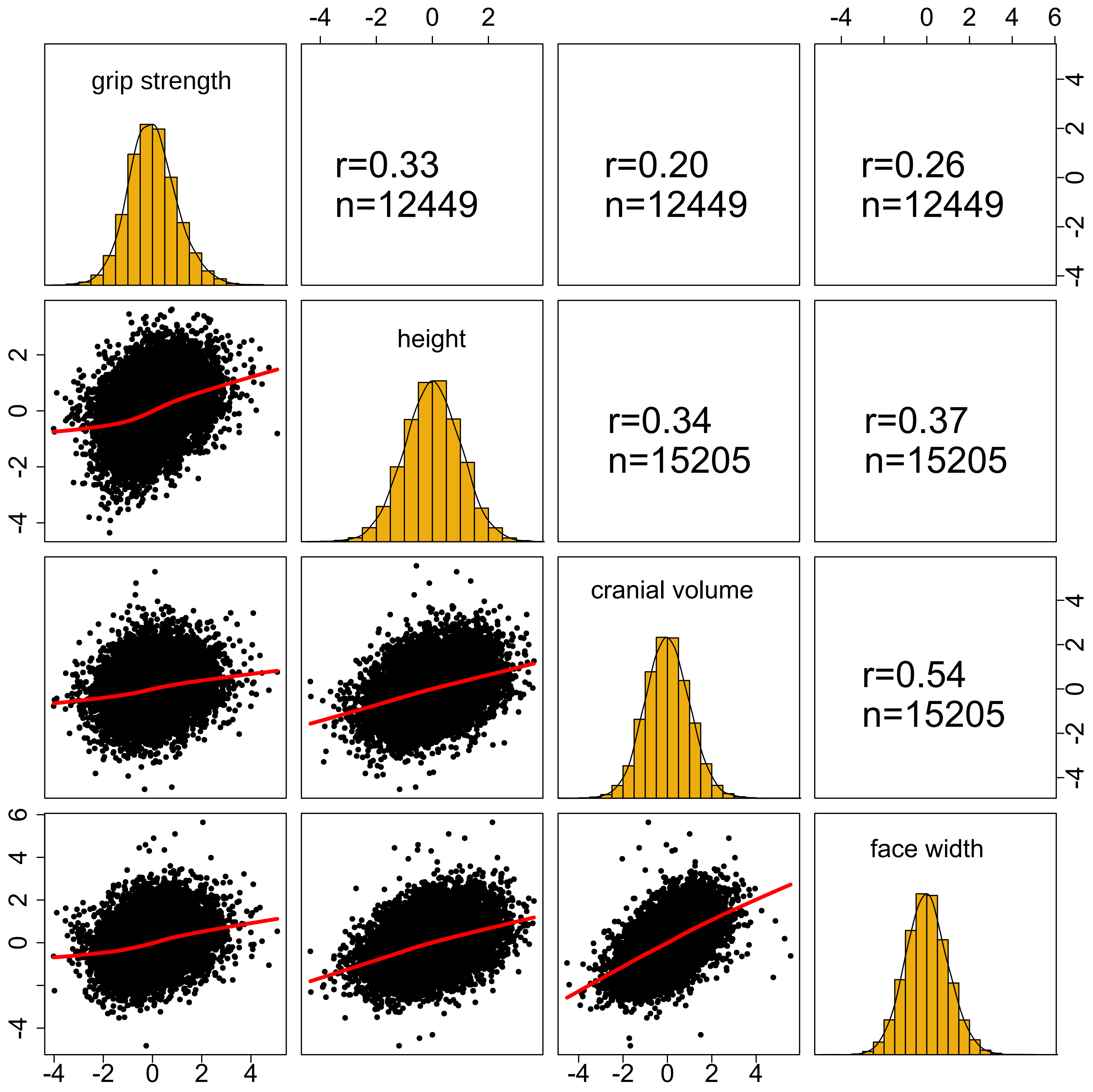

Figure S2. Pearson correlations between morphometric traits (residuals against age and birth date) for girls. Lines are fitted by loess smoothing.

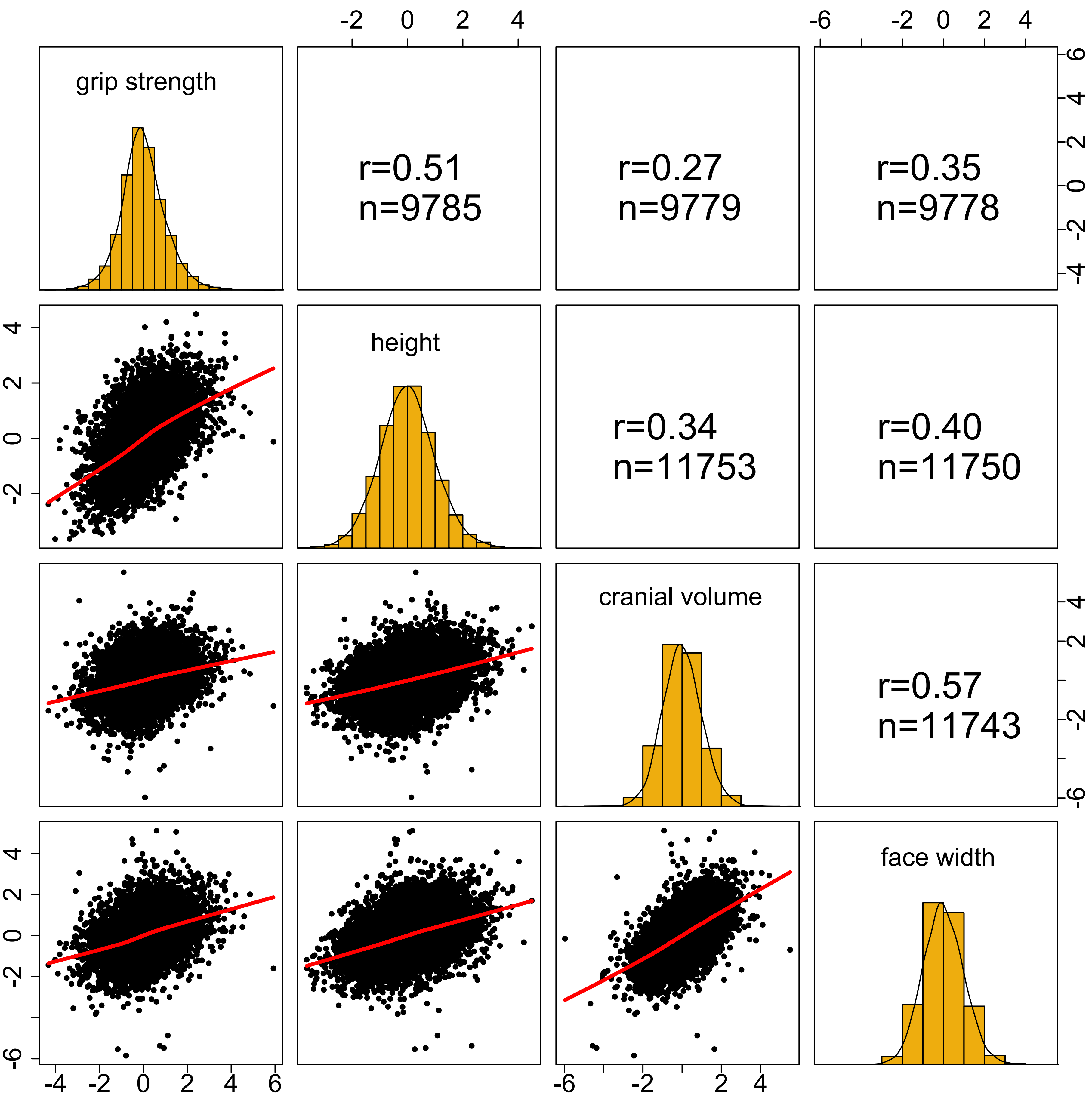

Figure S3. Pearson correlations between morphometric traits (residuals against age and birth date) for boys. Lines are fitted by loess smoothing.
