# Supplementary material for: Morphometric traits predict educational attainment independently of socioeconomic background
Source: BMC Public Health. 2019 Dec 18;19:1696. doi: 10.1186/s12889-019-8072-7 (PMC6921596; doi:10.1186/s12889-019-8072-7)
Supplement: Supplementary file 2 — Additional file 2. Electronic supplement 2. [file 12889_2019_8072_MOESM2_ESM.pdf]

Electronic supplement 2

Import data

```r
library(ormPlot)
#load the data from the package ormPlot
data(educ_data)
au<-educ_data

# center the year of birth to reduce the variance inflation factor
au$YOBc<-c(scale(au$YOB, scale = FALSE))
```

Run the main model

```r
#load the pakage
library(rms)

#calculate distribution summary values for each column in au dataframe
dd<-datadist(au, q.effect = c(0.5, 0.75) )

#add distribution summary values to subsequent model fits
options(datadist="dd")

#calculate the model fit
ocran<-orm(formula = educ_3 ~ Rural + sex + max_SEP_3 + n_siblings + cran_rzs +
             height_rzs + FW_rzs  + YOBc + (YOBc * sex) + (YOBc * Rural),
           data = au, y=TRUE, family=logistic, x=TRUE)
```

```r
ocran
```

```
## Logistic (Proportional Odds) Ordinal Regression Model
##
##  orm(formula = educ_3 ~ Rural + sex + max_SEP_3 + n_siblings +
##      cran_rzs + height_rzs + FW_rzs + YOBc + (YOBc * sex) + (YOBc *
##      Rural), data = au, x = TRUE, y = TRUE, family = logistic)
##
##                          Model Likelihood         Discrimination          Rank Discrim.
##                             Ratio Test                Indexes                Indexes
##  Obs          11032    LR chi2     2004.65    R2                    0.194    rho     0.407
##   1            1840    d.f.             11    g                     1.021
##   2            6250    Pr(> chi2) <0.0001    gr                    2.775
##   3            2942    Score chi2 1966.71    |Pr(Y>=median)-0.5| 0.333
##  Distinct Y       3    Pr(> chi2) <0.0001
##  Median Y         2
##  max |deriv| 6e-06
##
```

```
##                              Coef    S.E.   Wald Z Pr(>|Z|)
##   y>=2                        1.5760 0.0573  27.52 <0.0001
##   y>=3                       -1.4582 0.0568 -25.66 <0.0001
##   Rural=Rural                -0.5591 0.0428 -13.05 <0.0001
##   sex=Girl                    0.6370 0.0398  16.02 <0.0001
##   max_SEP_3=Skilled manual    0.4294 0.0509   8.43 <0.0001
##   max_SEP_3=Non-manual        1.2867 0.0502  25.61 <0.0001
##   n_siblings                 -0.1177 0.0127  -9.28 <0.0001
##   cran_rzs                    0.2102 0.0232   9.05 <0.0001
##   height_rzs                  0.1493 0.0215   6.95 <0.0001
##   FW_rzs                     -0.0851 0.0237  -3.60 0.0003
##   YOBc                       -0.0426 0.0080  -5.32 <0.0001
##   sex=Girl * YOBc             0.0215 0.0088   2.45 0.0141
##   Rural=Rural * YOBc          0.0646 0.0094   6.91 <0.0001
##
```

Verify model assumptions

```
#using the suggested method for assesing ordinal proportional odds model assumptions
# see Guisan, Antoine, and Frank E. Harrell. 2000.
#"Ordinal Response Regression Models in Ecology."
#Journal of Vegetation Science 11 (5): 617-26. https://doi.org/10.2307/3236568.

au_yob_factor<-au
#to avoid singular information matrix in lrm.fit for YOB
au_yob_factor$YOB<-as.factor(au_yob_factor$YOB)

plot.xmean.ordinaly(educ_3 ~ Rural + sex + max_SEP_3 + n_siblings + cran_rzs +
                      height_rzs + FW_rzs + YOB,  data = au_yob_factor, topcats = 5)
```

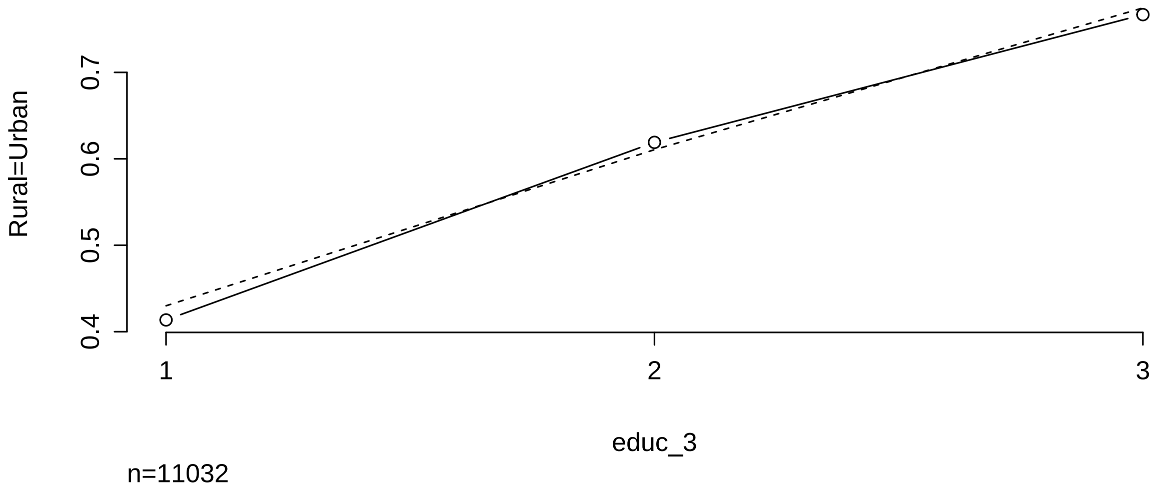

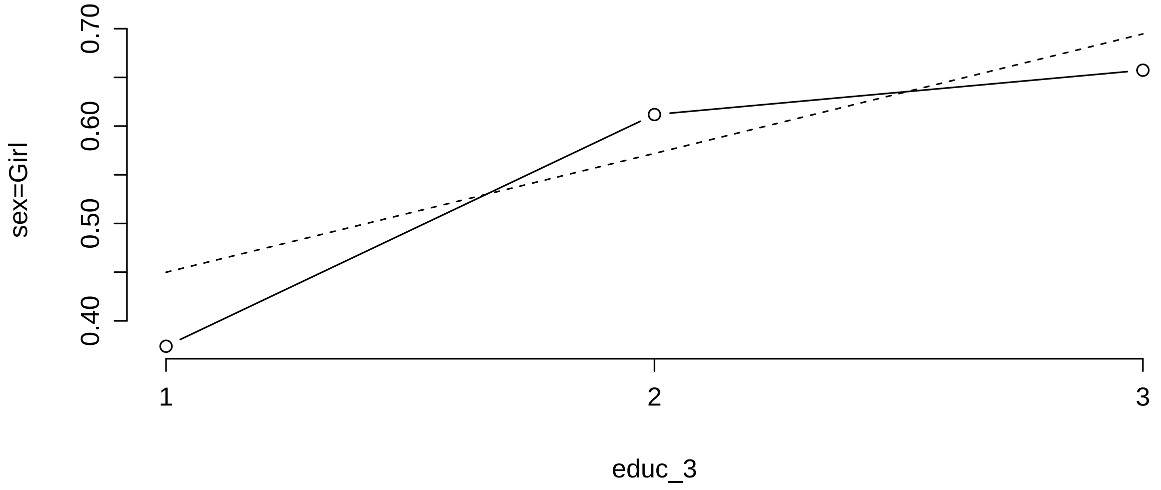

n=11032

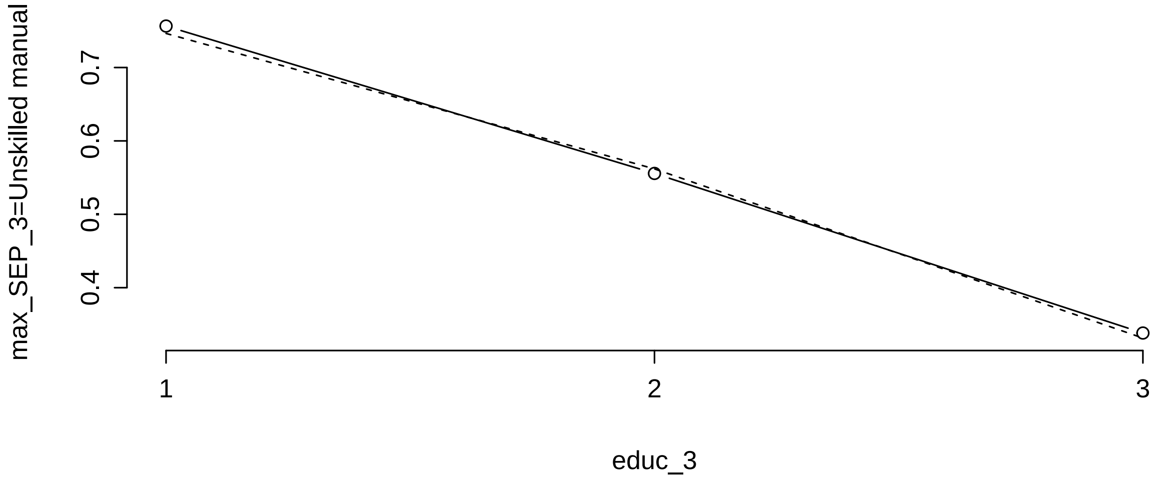

n=11032

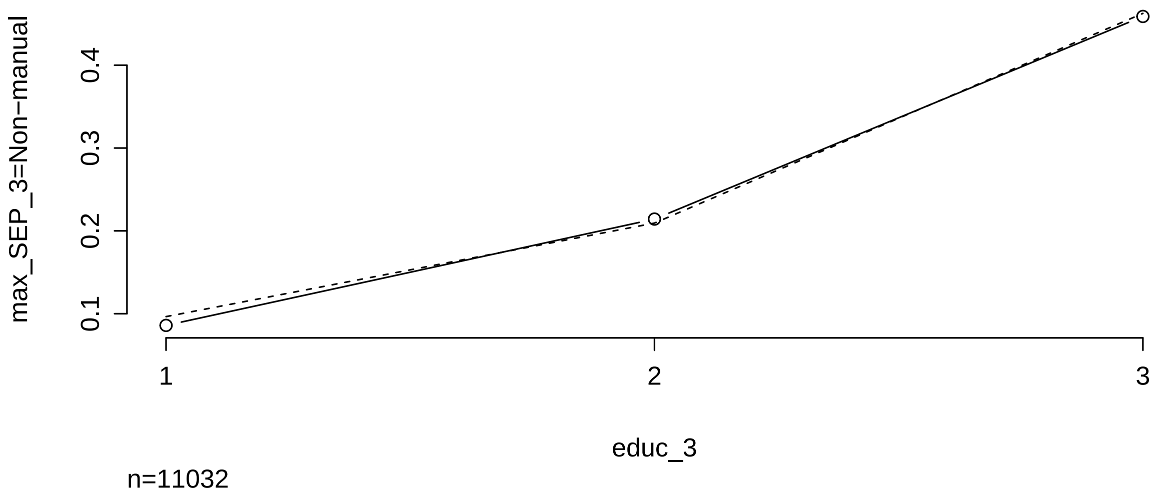
max_SEP_3=Non−manual
0.1
0.2
0.3
0.4
1
2
3
educ_3
n=11032

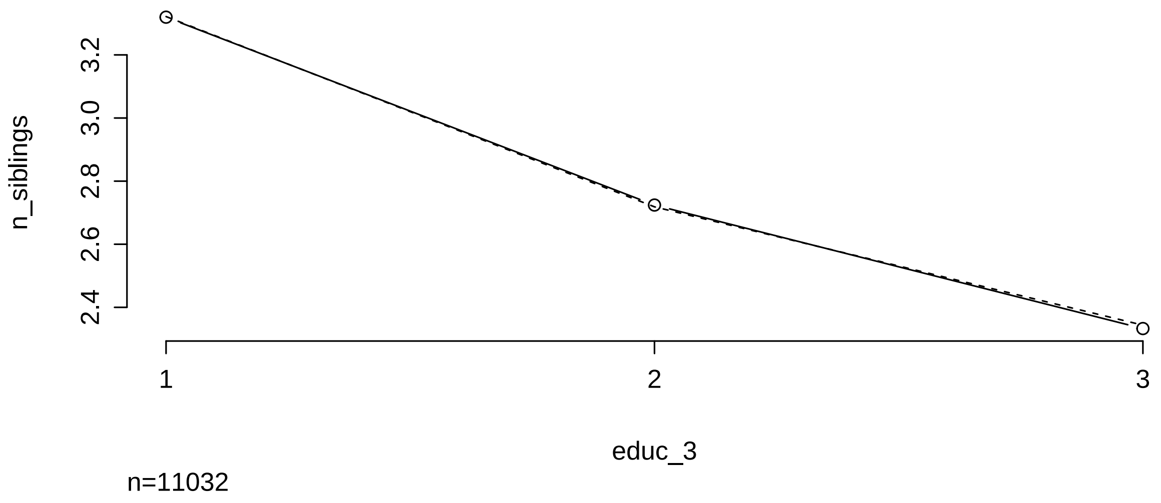
n_siblings
2.4
2.6
2.8
3.0
3.2
1
2
3
educ_3
n=11032

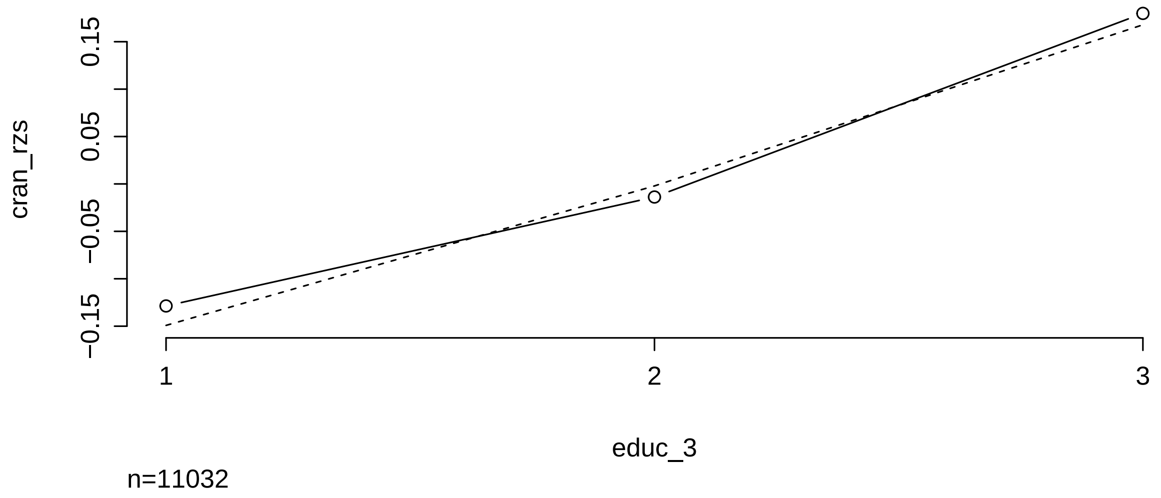
cran_rzs
0.15
0.05
−0.05
−0.15
1
2
3
educ_3
n=11032

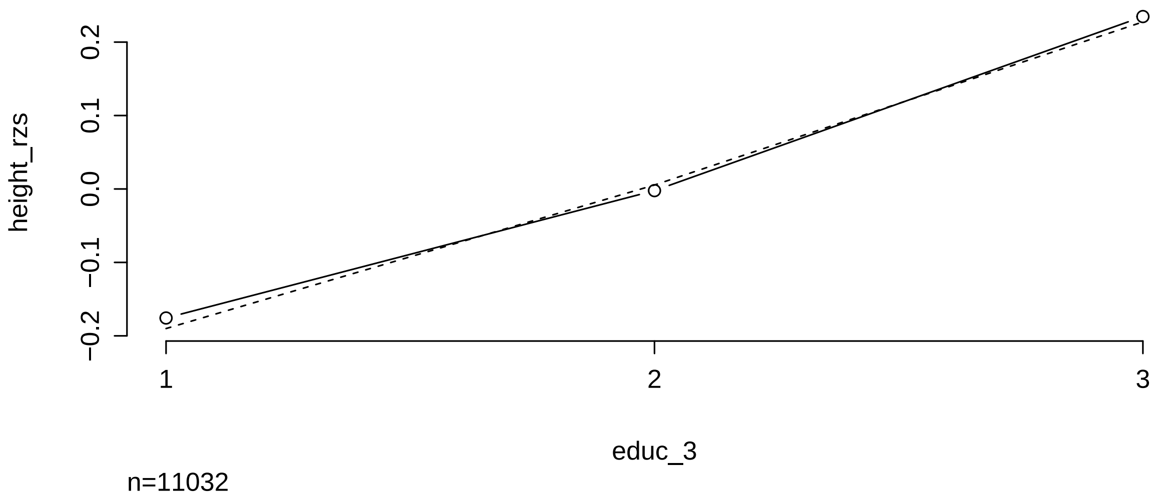
height_rzs
0.2
0.1
0.0
−0.1
−0.2
1
2
3
educ_3
n=11032

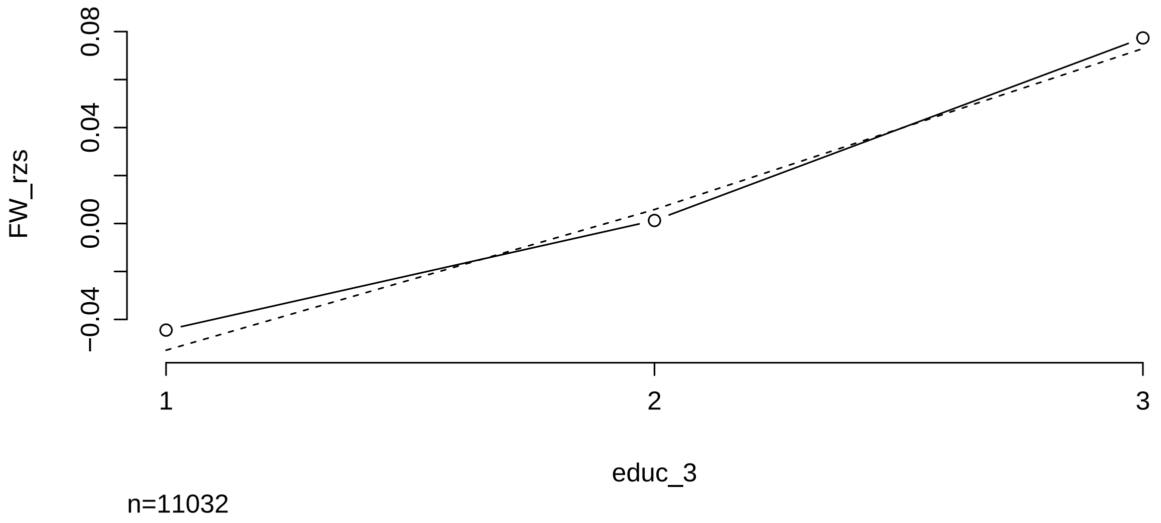
FW_rzs
−0.04
0.00
0.04
0.08
1
2
3
educ_3
n=11032

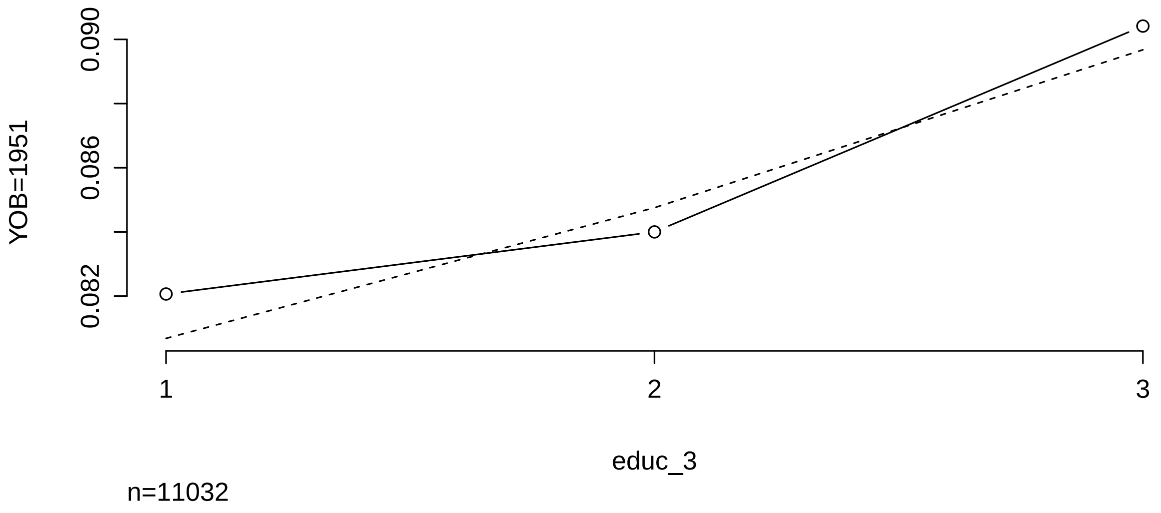
YOB=1951
0.082
0.086
0.090
1
2
3
educ_3
n=11032

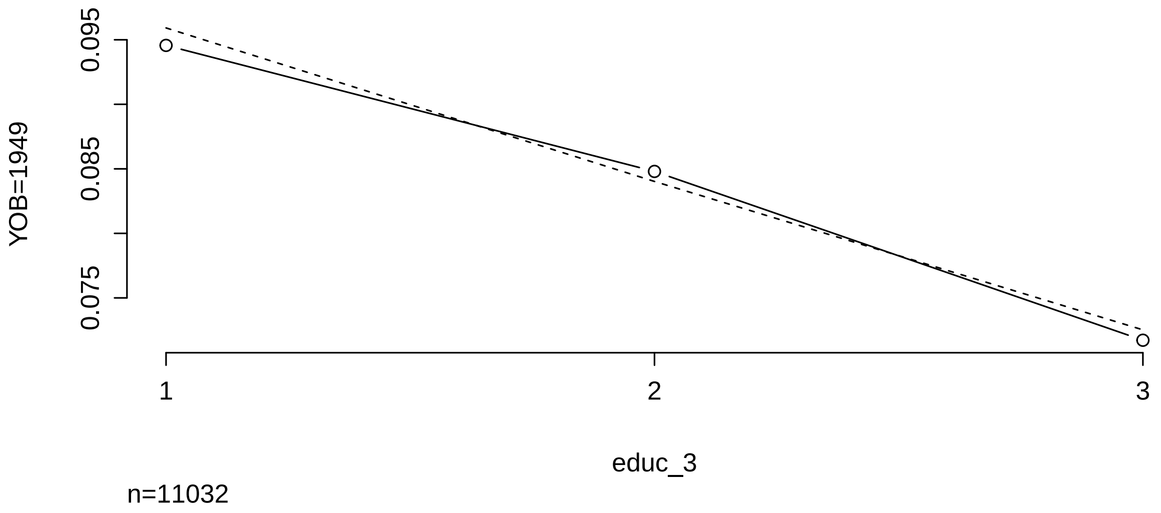
YOB=1949
0.095
0.085
0.075
1
2
3
educ_3
n=11032

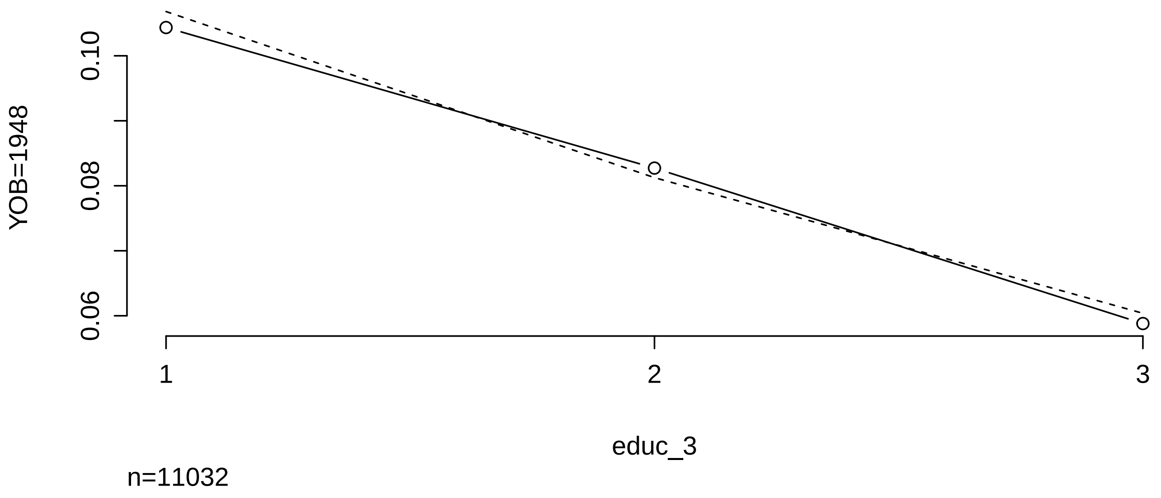
YOB=1948
0.10
0.08
0.06
1
2
3
educ_3
n=11032

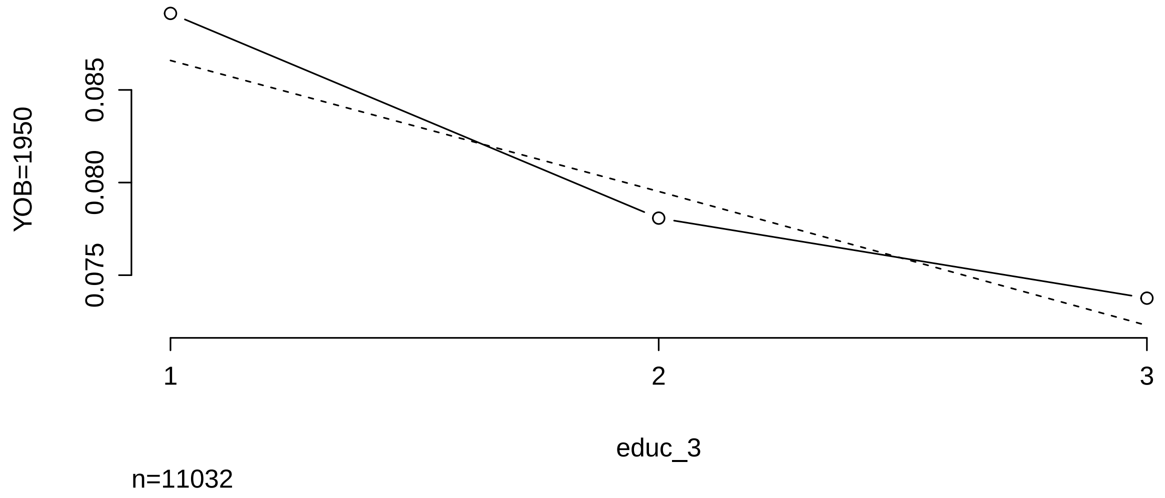

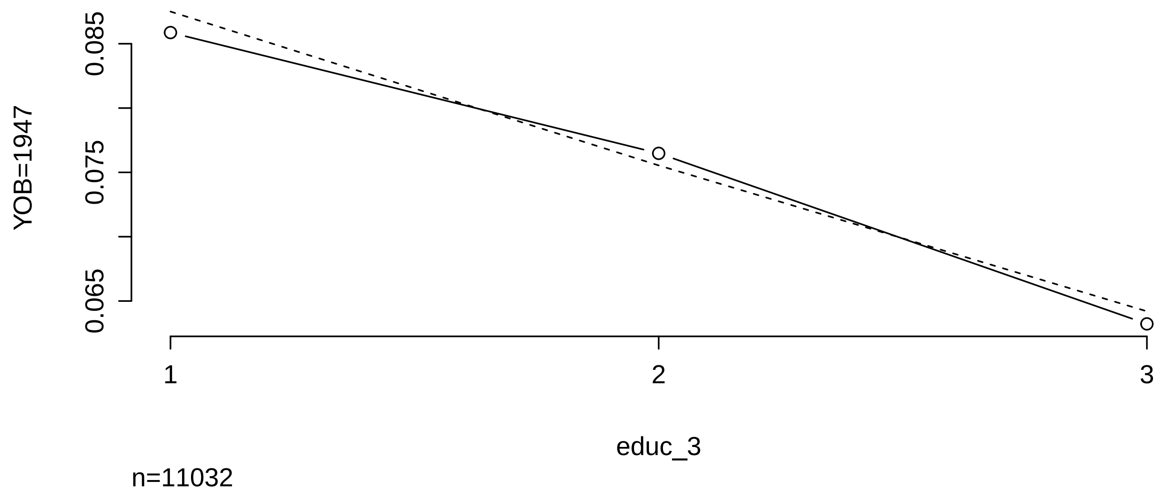

Solid lines connect the actual means while the dashed lines connect the expected means. The ordinalitty assumption holds for all variables, with sex deviating the most from perfect fit.

```
round(vif(ocran),2)
```

```
##                 sex=Girl max_SEP_3=Skilled manual     max_SEP_3=Non-manual
##                     1.01                     1.18                     1.21
##               n_siblings                 cran_rzs               height_rzs
##                     1.05                     1.45                     1.21
##                   FW_rzs                     YOBc           sex=Girl * YOBc
##                     1.51                     4.04                     3.27
##       Rural=Rural * YOBc
##                     1.42
```

No significant collinearity can be found in the model terms (all varaince inflation factors <5).

Plot the ordinal regression model

Plot summary

```
mytheme<-theme_classic() + theme(text = element_text(size = 12, colour = "black"))
orm_summary_forestplot<-forestplot(summary(ocran), return_ggplots = F,
                                   theme = mytheme,  plot.widths = c(0.6,0.4),
                                   row.names.y = c("Number of children",
                                                   "Cranial volume",
                                                   "Height", "Face width",
                                                   "Birth year",
                                                   "Origin: rural vs urban",
                                                   "Sex: boys vs girls ",
                                                   "SEP: manual skilled vs unskilled",
                                                   "SEP: non-manual vs unskilled"))
```

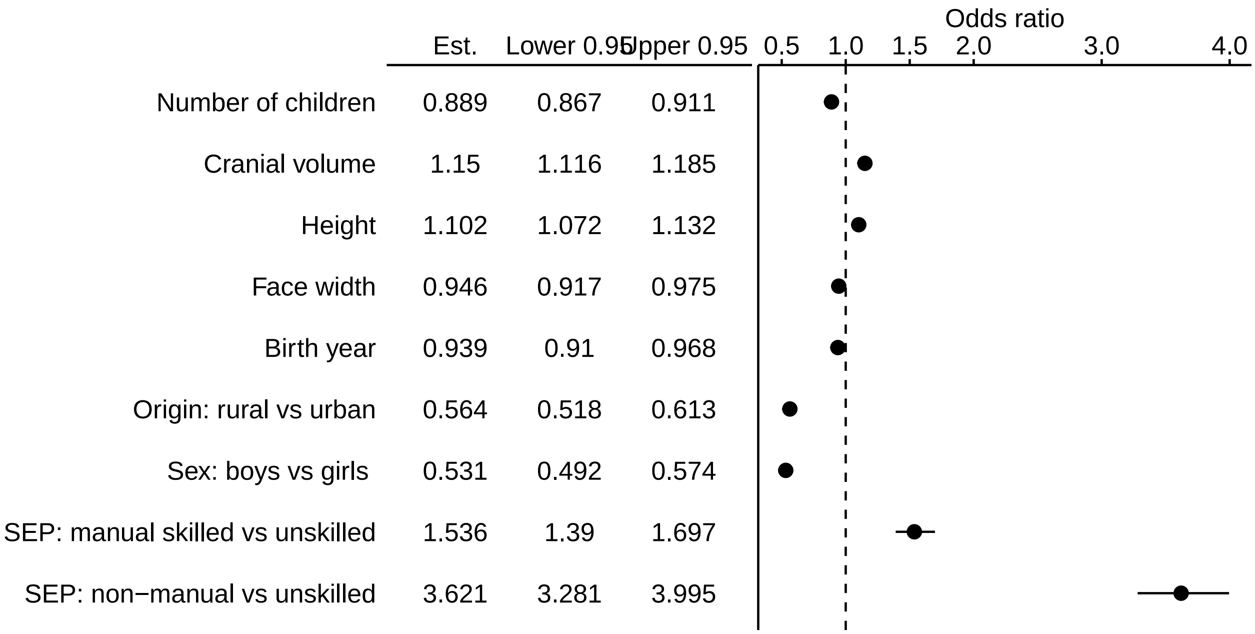

Plot predictions

```
#setting the general look of graphs
colors <- c("#4a9878", "#0a191e", "#d8b65c")
colors <- c("red", "#0a191e", "darkgoldenrod2")
educ_names <- c("Primary", "Secondary", "Tertiary")
```

Cranial volume

```
p<-plot(ocran, cran_rzs,  max_SEP_3, c(Rural, sex),
                          xlab = "Cranial volume (residuals to age an birth date)",
                          facet_labels = list(Rural = c("Urban","Rural"),
                                              sex=c("Boys","Girls"),
                                              max_SEP_3=c("Unskilled manual",
```

```
                                                          "Skilled manual",
                                                          "Non-manual")))
```

```
final_plot<-p + labs(subtitle = "Highest parental profession",
                     color = "Education", fill = "Education",
                     y ="Propability of obtaining") +
  theme(plot.subtitle = element_text(hjust = 0.5),
        legend.direction = "horizontal",
        legend.position = "bottom",
        legend.text = theme_get()$text,
        legend.title=element_blank(),
        strip.background = element_rect(linetype="blank", fill = "white"),
        strip.text = theme_get()$text) +
  coord_fixed(ratio = 8) +
  scale_color_manual(values = colors, labels = educ_names) +
  scale_fill_manual(values = colors, labels = educ_names)

final_plot
```

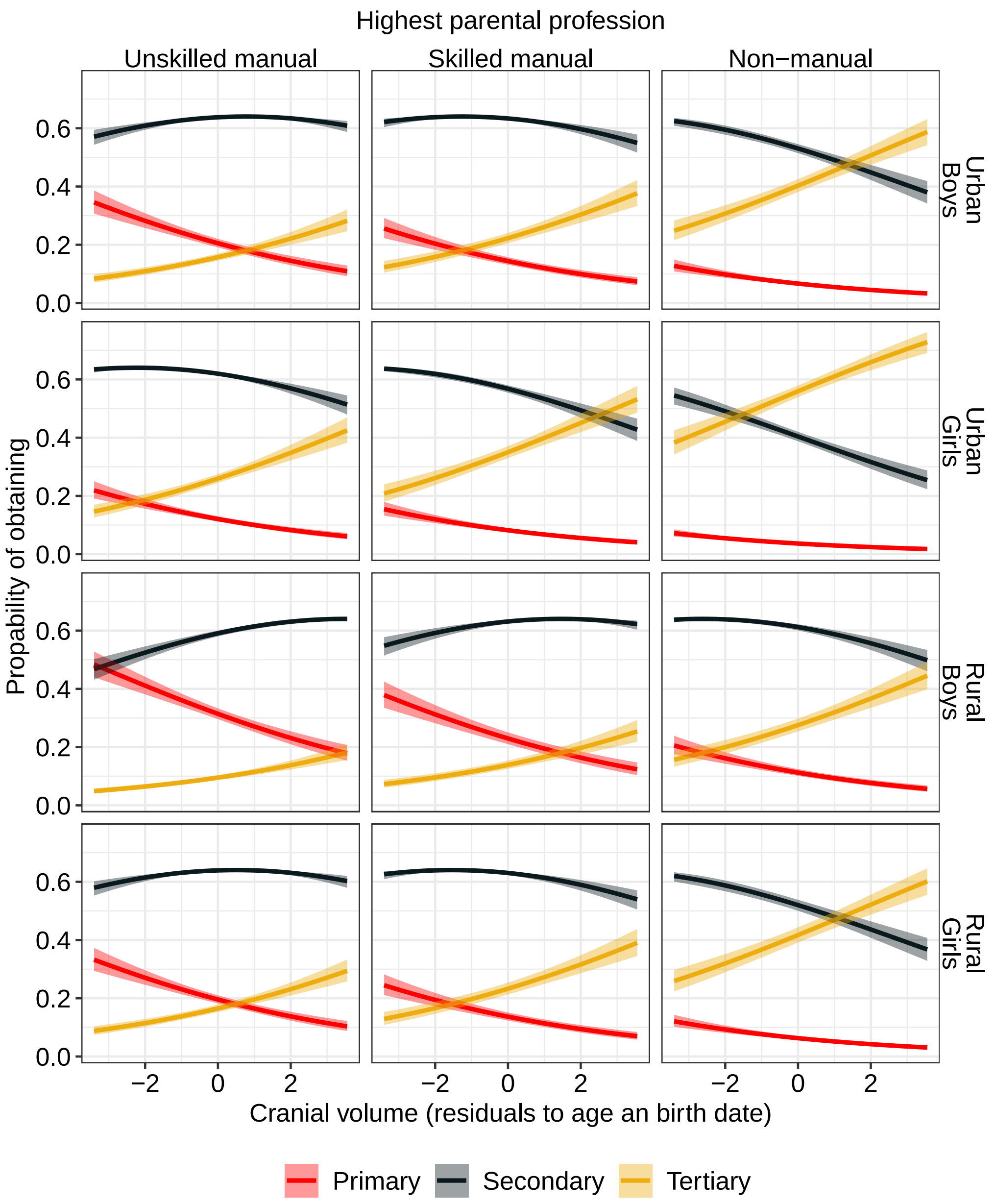

Height

```
educ_height<-plot(ocran, height_rzs,  max_SEP_3, c(Rural, sex),
                  xlab = "Height (residuals to age an birth date)",
                  facet_labels = list(Rural = c("Urban", "Rural"),
                                      sex=c("Boys","Girls"),
                                      max_SEP_3=c("Unskilled manual",
                                                  "Skilled manual",
                                                  "Non-manual")))

educ_height <-educ_height + labs(subtitle = "Highest parental profession",
                       color = "Education", fill = "Education",
```

```
                                    y ="Propability of obtaining") +
  theme(plot.subtitle = element_text(hjust = 0.5),
        legend.direction = "horizontal",
        legend.position = "bottom",
        legend.text = theme_get()$text,
        legend.title=element_blank(),
        strip.background = element_rect(linetype="blank", fill = "white"),
        strip.text = theme_get()$text) +
  coord_fixed(ratio = 8) +
  scale_color_manual(values = colors, labels = educ_names) +
  scale_fill_manual(values = colors, labels = educ_names)

educ_height
```

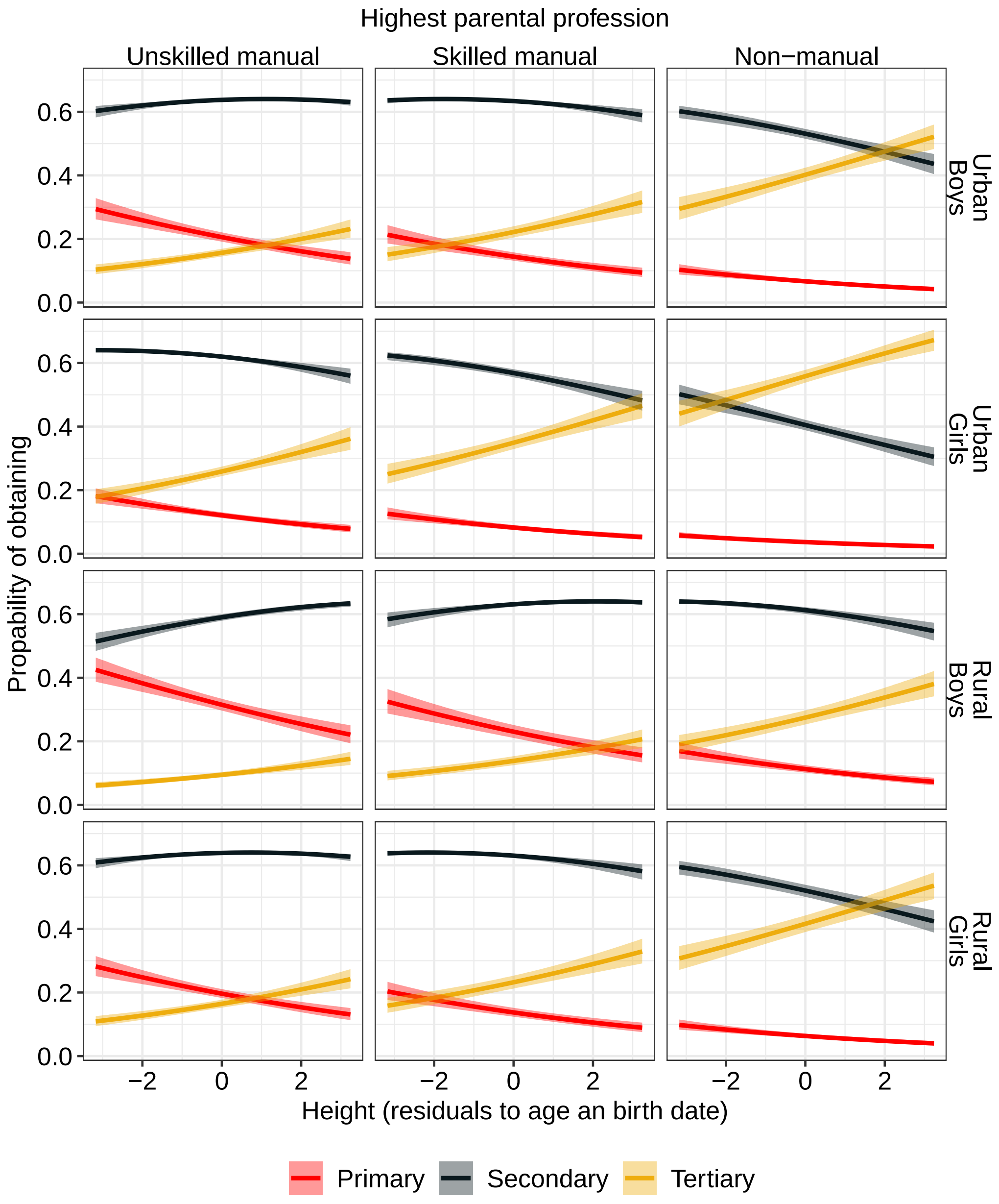

Face Width

```
educ_fw<-plot(ocran, FW_rzs,  max_SEP_3, c(Rural, sex),
                          xlab = "Face Width (residuals to age an birth date)",
                          facet_labels = list(Rural = c("Urban", "Rural"),
                                              sex=c("Boys","Girls"),
                                              max_SEP_3=c("Unskilled manual",
                                                          "Skilled manual",
                                                          "Non-manual")))


educ_fw <-  educ_fw + labs(subtitle = "Highest parental profession",
                           color = "Education", fill = "Education",
                           y ="Propability of obtaining") +
  theme(plot.subtitle = element_text(hjust = 0.5),
        legend.direction = "horizontal",
        legend.position = "bottom",
        legend.text = theme_get()$text,
        legend.title=element_blank(),
        strip.background = element_rect(linetype="blank", fill = "white"),
        strip.text = theme_get()$text) +
  coord_fixed(ratio = 8) +
  scale_color_manual(values = colors, labels = educ_names) +
  scale_fill_manual(values = colors, labels = educ_names)

educ_fw
```

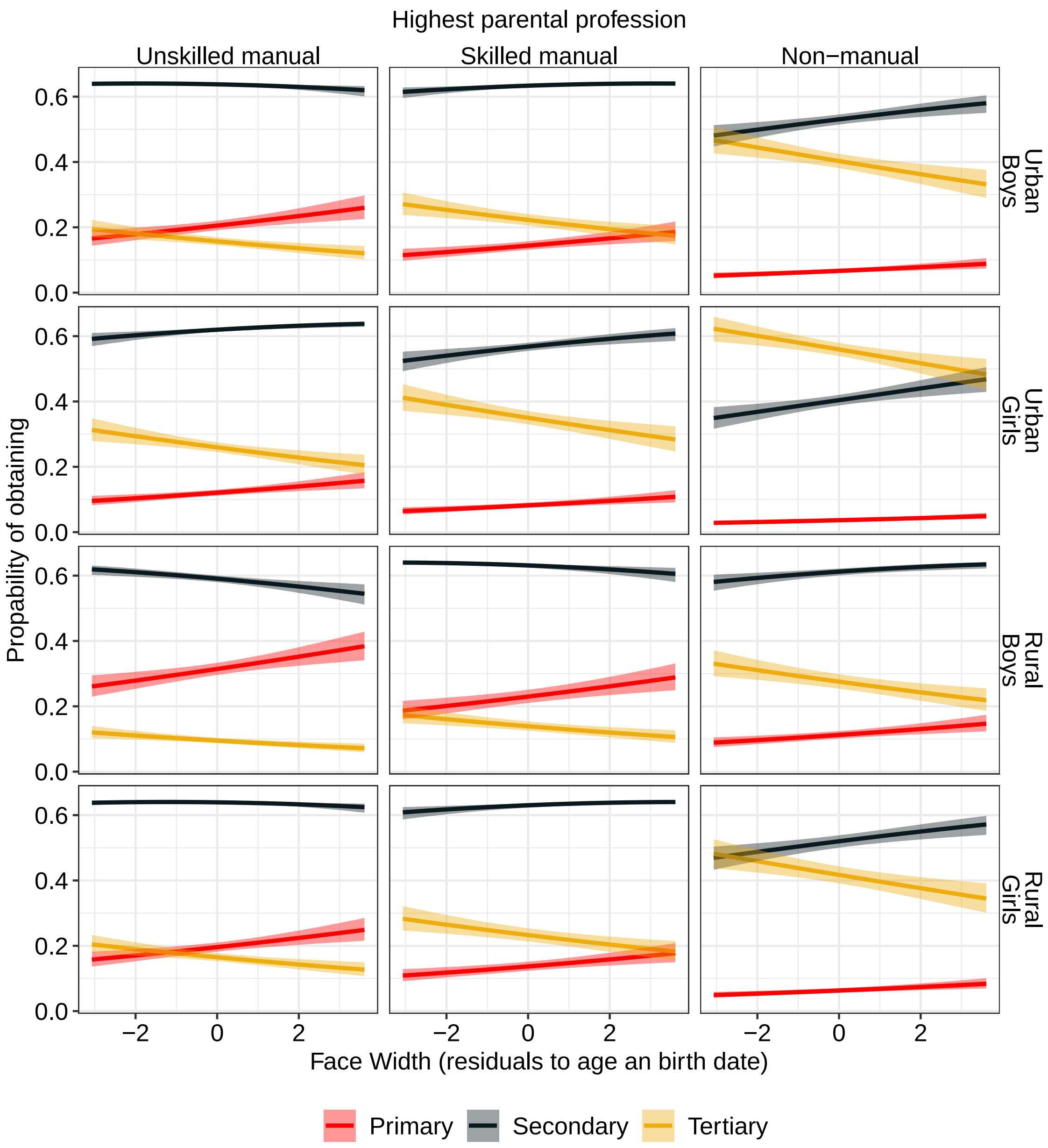

Logistic regression models to compare 2 educations

Secondary vs tertiary education model

```
edu23_model<-lrm(formula = educ_3 ~ Rural + sex + max_SEP_3 + n_siblings +
                   cran_rzs + height_rzs + FW_rzs + YOBc + (sex*YOBc),
                 data = au, subset=(educ_3!=1),   y=TRUE, x=TRUE)
```

```
edu23_model
```

```
## Logistic Regression Model
##
```

```
##  lrm(formula = educ_3 ~ Rural + sex + max_SEP_3 + n_siblings +
##      cran_rzs + height_rzs + FW_rzs + YOBc + (sex * YOBc), data = au,
##      subset = (educ_3 != 1), x = TRUE, y = TRUE)
##
##                        Model Likelihood     Discrimination    Rank Discrim.
##                           Ratio Test           Indexes           Indexes
##  Obs           9192    LR chi2     914.34    R2       0.133    C       0.691
##   2            6250    d.f.            10    g        0.808    Dxy     0.383
##   3            2942    Pr(> chi2) <0.0001    gr       2.244    gamma   0.383
##  max |deriv| 2e-07                           gp       0.165    tau-a   0.167
##                                              Brier    0.196
##
##                           Coef    S.E.   Wald Z Pr(>|Z|)
##  Intercept                -1.0141 0.0720 -14.09 <0.0001
##  Rural=Rural              -0.4040 0.0552  -7.32 <0.0001
##  sex=Girl                  0.2172 0.0500   4.34 <0.0001
##  max_SEP_3=Skilled manual  0.2909 0.0633   4.59 <0.0001
##  max_SEP_3=Non-manual      1.1422 0.0567  20.13 <0.0001
##  n_siblings               -0.0933 0.0182  -5.12 <0.0001
##  cran_rzs                  0.2055 0.0289   7.11 <0.0001
##  height_rzs                0.1401 0.0266   5.27 <0.0001
##  FW_rzs                   -0.0759 0.0294  -2.58 0.0099
##  YOBc                     -0.0773 0.0092  -8.41 <0.0001
##  sex=Girl * YOBc           0.0568 0.0107   5.30 <0.0001
##
```

```
mytheme<-theme_classic() + theme(text = element_text(size = 12))
plots<-forestplot(summary(edu23_model), limits = c(0.25,3.6), digits = 2,
                  theme = mytheme,
                  row.names.y = c("Number of children","Cranial volume",
                                  "Height", "Face width", "Birth year",
                                  "Origin: rural vs urban", "Sex: boys vs girls ",
                                  "SEP: manual skilled vs unskilled",
                                  "SEP: non-manual vs unskilled"),
                  return_ggplots=T)


educ23_joined_plot<-join_ggplots(plots[[1]],plots[[2]], plot.widths = c(0.65,0.35))
```

	Est.	Lower 0.95	Upper 0.95
Number of children	0.91	0.88	0.94
Cranial volume	1.15	1.1	1.19
Height	1.1	1.06	1.13
Face width	0.95	0.92	0.99
Birth year	0.94	0.91	0.97
Origin: rural vs urban	0.67	0.6	0.74
Sex: boys vs girls	0.82	0.74	0.9
SEP: manual skilled vs unskilled	1.34	1.18	1.51
SEP: non−manual vs unskilled	3.13	2.8	3.5

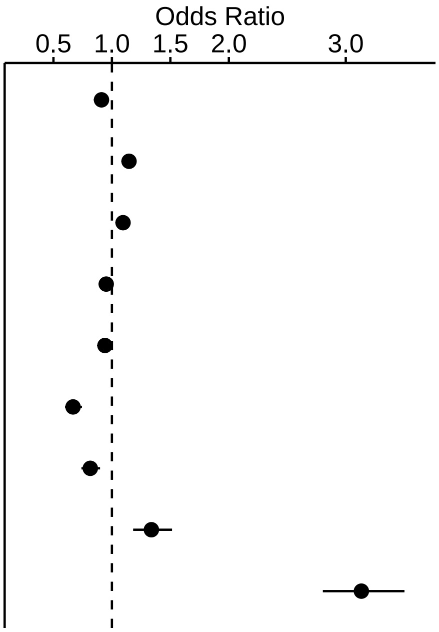

Primary vs secondary education model

```
edu12_model<-lrm(formula = educ_3 ~ Rural + sex + max_SEP_3 + n_siblings +
                   cran_rzs + height_rzs +  YOBc  + (Rural * YOBc) +
                   (Rural * sex) + sex*cran_rzs, data = au, subset=(educ_3!=3),
                 y=TRUE, x=TRUE)
edu12_model
```

```
## Logistic Regression Model
##
##  lrm(formula = educ_3 ~ Rural + sex + max_SEP_3 + n_siblings +
##      cran_rzs + height_rzs + YOBc + (Rural * YOBc) + (Rural *
##      sex) + sex * cran_rzs, data = au, subset = (educ_3 != 3),
##      x = TRUE, y = TRUE)
##
##                        Model Likelihood     Discrimination    Rank Discrim.
##                           Ratio Test           Indexes           Indexes
##  Obs           8090    LR chi2     872.30    R2       0.155    C       0.719
##   1            1840    d.f.            11    g        0.986    Dxy     0.437
##   2            6250    Pr(> chi2) <0.0001    gr       2.679    gamma   0.437
##  max |deriv| 3e-11                           gp       0.153    tau-a   0.154
##                                              Brier    0.157
##
##                           Coef    S.E.   Wald Z Pr(>|Z|)
##  Intercept                 1.0370 0.0766 13.54  <0.0001
##  Rural=Rural              -0.3626 0.0767 -4.73  <0.0001
##  sex=Girl                  1.1213 0.0841 13.33  <0.0001
##  max_SEP_3=Skilled manual  0.4510 0.0763  5.91  <0.0001
##  max_SEP_3=Non-manual      0.9587 0.0944 10.15  <0.0001
##  n_siblings               -0.1018 0.0161 -6.34  <0.0001
##  cran_rzs                  0.0607 0.0388  1.57  0.1175
##  height_rzs                0.0899 0.0306  2.93  0.0033
```

```
##   YOBc                          0.0166 0.0087  1.90  0.0571
##   Rural=Rural * YOBc            0.0569 0.0131  4.35  <0.0001
##   Rural=Rural * sex=Girl       -0.2815 0.1142 -2.46  0.0137
##   sex=Girl * cran_rzs           0.1202 0.0572  2.10  0.0356
##
```

```r
mytheme2<-theme_classic() + theme(text = element_text(color = "red", size = 12),
                                  line = element_line(color= "red"),
                                  rect = element_rect(color="red"))

plots_educ_12<-forestplot(summary(edu12_model), limits = c(0.25,3.6),
                          shape = 17 ,
                          digits = 2,
                          theme=mytheme2,
                          row.names.y = c("Number of children","Cranial volume",
                                          "Height", "Birth year",
                                          "Origin: rural vs urban",
                                          "Sex: boys vs girls ",
                                          "SEP: manual skilled vs unskilled",
                                          "SEP: non-manual vs unskilled"),
                          return_ggplots=T)
p2<-plots_educ_12[[2]]
p1<-plots_educ_12[[1]]
educ12_joined<-join_ggplots(p1,p2, plot.widths = c(0.65,0.35))
```

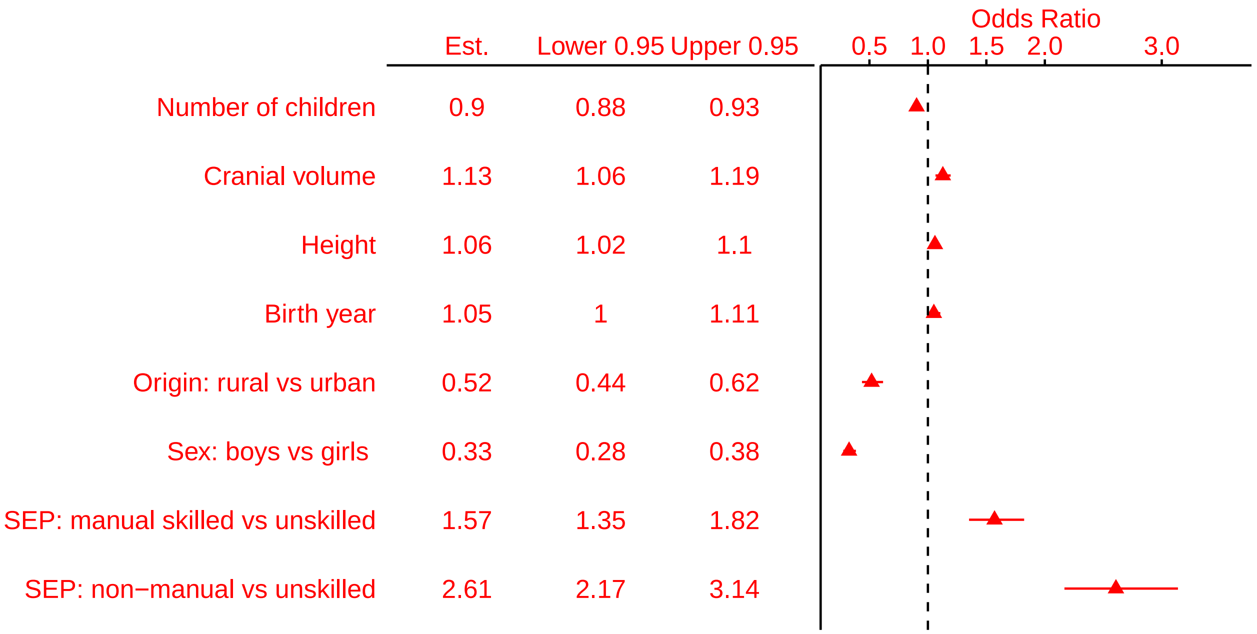

Plot SEP count by education

```r
# New facet label names
levels(au$Rural) <- c("Urban", "Rural")
levels(au$sex) <- c("Boys","Girls")
levels(au$educ_3) <- educ_names
```

```r
sep_count_plot<-ggplot(au, aes(x= max_SEP_3, color= educ_3, fill= educ_3)) +
  geom_bar( stat="count") +
  facet_grid(Rural ~ sex) +
  theme_light() +
  scale_fill_manual (values=c("red", "black", "darkgoldenrod2"),name="Education") +
  scale_color_manual (values=c("red", "black", "darkgoldenrod2"), name="Education") +
  labs(x = "Parental socioeconomic position (SEP)", y = "Number of observations")

sep_count_plot<-sep_count_plot + theme(
        text = element_text(size = 12),
        axis.text = element_text(size = 12, colour = "black"),
        legend.text = element_text(size = 12, colour = "black"),
        axis.text.x = element_text(angle=45, hjust=1),
        #legend.title=element_blank(),
        strip.background = element_rect(linetype="blank", fill = "white"),
        strip.text = element_text(size = 12, colour = "black"))
sep_count_plot
```

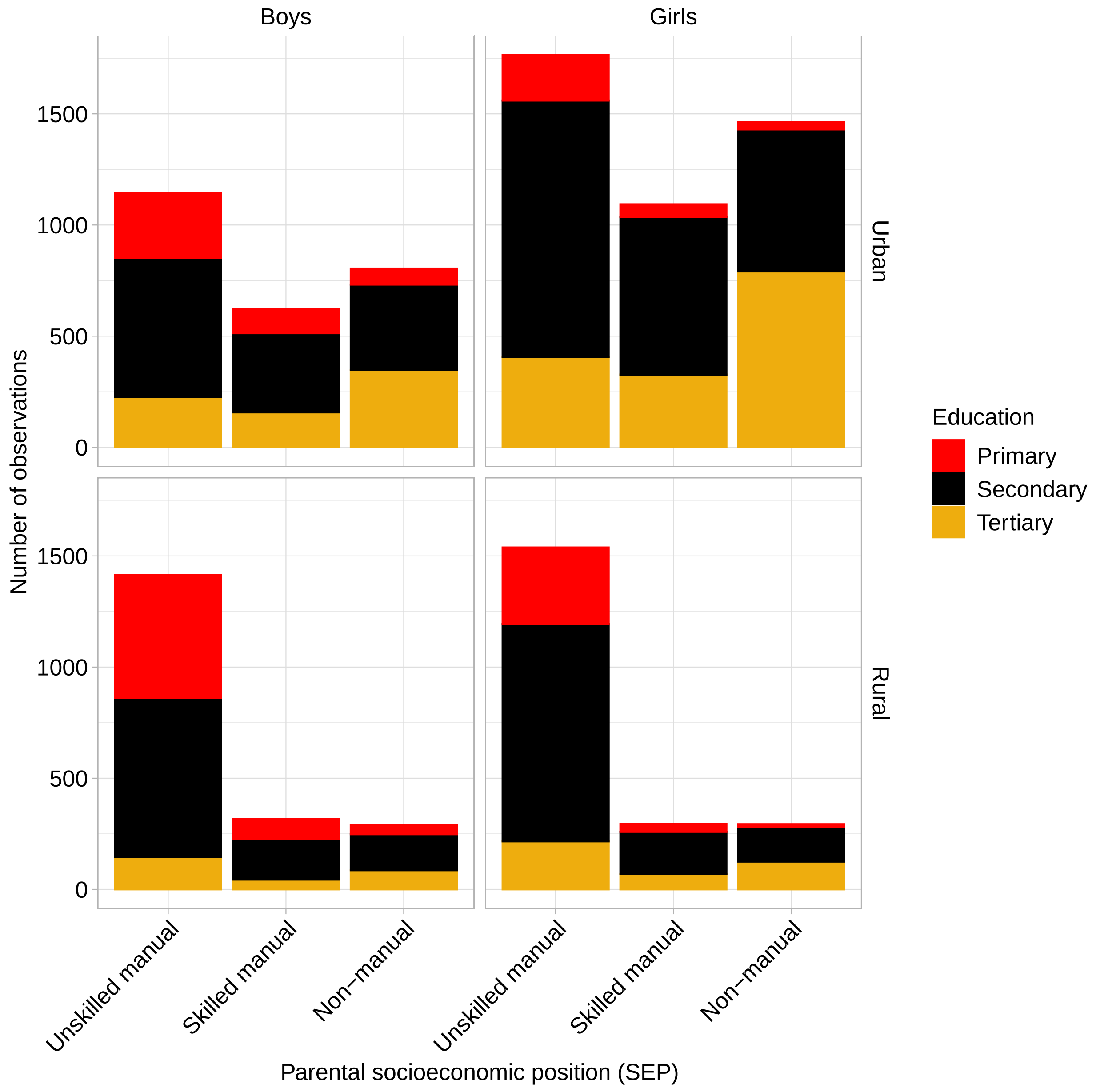
Boys
Girls
Urban
Rural
Number of observations
0
500
1000
1500
Unskilled manual
Skilled manual
Non–manual
Parental socioeconomic position (SEP)
Education
Primary
Secondary
Tertiary
